# A snapshot of pneumonia research activity and collaboration patterns (2001–2015): a global bibliometric analysis

**DOI:** 10.1186/s12874-019-0819-4

**Published:** 2019-09-05

**Authors:** José M. Ramos-Rincón, Héctor Pinargote-Celorio, Isabel Belinchón-Romero, Gregorio González-Alcaide

**Affiliations:** 1 Department of Internal Medicine, General University Hospital of Alicante, calle Pintor Baeza, 12, 03010 Alicante, Spain; 2 Department of Clinical Medicine, Miguel Hernandez University of Elche de Elche, Sant Joan d’Alacant, Alicante, Spain; 3 Service of Dermatology, General University Hospital of Alicante, Alicante, Spain; 4 Department of History of Science and Documentation, University of Valencia, Valencia, Spain

**Keywords:** Pneumonia, Bibliometrics, Scientometrics, Scientific production, Mapping, Publications

## Abstract

**Background:**

This article describes a bibliometric review of the scientific production, geographical distribution, collaboration, impact, and subject area focus of pneumonia research indexed on the Web of Science over a 15-year period.

**Methods:**

We searched the Web of Science database using the Medical Subject Heading (MeSH) of “Pneumonia” from January 1, 2001 to December 31, 2015. The only document types we studied were original articles and reviews, analyzing descriptive indicators by five-year periods and the scientific production by country, adjusting for population, economic, and research-related parameters.

**Results:**

A total of 22,694 references were retrieved. The number of publications increased steadily over time, from 981 publications in 2001 to 1977 in 2015 (R^2^ = 0.956). The most productive country was the USA (38.49%), followed by the UK (7.18%) and Japan (5.46%). Research production from China increased by more than 1000%. By geographical area, North America (42.08%) and Europe (40.79%) were most dominant. Scientific production in low- and middle-income countries more than tripled, although their overall contribution to the field remained limited (< 15%).

Overall, 18.8% of papers were the result of an international collaboration, although this proportion was much higher in sub-Saharan Africa (46.08%) and South Asia (23.43%). According to the specific MeSH terms used, articles focused mainly on “Pneumonia, Bacterial” (19.99%), followed by “Pneumonia, Pneumococcal” (7.02%) and “Pneumonia, Ventilator-Associated” (6.79%).

**Conclusions:**

Pneumonia research increased steadily over the 15-year study period, with Europe and North America leading scientific production. About a fifth of all papers reflected international collaborations, and these were most evident in papers from sub-Saharan Africa and South Asia.

**Electronic supplementary material:**

The online version of this article (10.1186/s12874-019-0819-4) contains supplementary material, which is available to authorized users.

## Background

Pneumonia is an important infectious disease worldwide and is associated with high morbidity, mortality and health system expenditure [1, 2]. In 2015, data from the Global Burden of Disease study showed that lower respiratory tract infections, including pneumonia, were the third most common cause of death, exceeded only by ischemic heart disease and cerebrovascular disease [3]. Community-acquired pneumonia (CAP) remains the primary cause of death from infectious disease globally, and its high impact on morbidity and mortality is especially concentrated in children under five and the elderly [1, 4–6]. The World Health Organization (WHO) predicted that deaths from lower respiratory tract infections would remain among the top four causes of deaths up to at least 2030 [7]. Antibiotic-resistant strains have also been on the rise, although resistance does not appear to be related to mortality. However, pneumonia is associated with high rates of hospitalization and length of hospital stay. Moreover, it has considerable long-term effects on quality of life, and long-term prognosis is worse in patients with pneumococcal pneumonia [1].

Despite the public health importance of the disease, few studies have evaluated research in the area using bibliometric methods. Indeed, only Head et al. (2015) have analyzed publications on pneumonia, and their work was limited in geographical scope to the UK [8, 9]. In this study, by analyzing scientific papers on pneumonia published in the main international scientific journals, we aimed to identify the scientific contribution of different countries to the worldwide research effort, the most cited landmark articles, the degree and nature of scientific collaboration, and the topics addressed.

This bibliometric description can provide relevant information for researchers in the field, particularly new scientists, giving a snapshot of strong research areas in pneumonia and global health as well as possible gaps requiring additional investments [10–12]. The paper also provides clues for addressing the weaknesses observed, such as the need to promote North-South collaborations and other research initiatives with countries that have relatively little scientific development on the topic [9, 13].

The aim of the present study is to assess the scientific literature on pneumonia that is indexed in the Web of Science (WoS). Specifically, we will analyze: (1) the evolution of scientific production; (2) its distribution by countries and regions; (3) the impact of the research papers; and (4) the degree of international collaboration. Finally, we will present details on the subject area focus of different publications according to the Medical Subject Headings (MeSH).

## Methods

### Identifying the population of study documents

For the performance of the study, we opted to identify documents about pneumonia by means of the MeSH thesaurus in the MEDLINE database because this is a detailed instrument for controlled terminology. The thesaurus employs both a human team of specialist indexers to analyze each article and assign medical subject headings to it, plus automated processes to improve indexing; the result is a highly consistent system of classification for research topics [14–16]. The pneumonia descriptor was introduced in 1963 as a disease of the respiratory tract and the lung, and it was defined as “infection of the lung often accompanied by inflammation” [17]. Synonyms of this descriptor (and therefore also included in search results) are “Lung Inflammation” and “Pulmonary Inflammation”. Additional file 1: Table S1 shows the MeSH tree structure for “Pneumonia”.

The next step was to identify the documents assigned with the MEDLINE descriptor of “pneumonia” indexed in the WoS. This body of research constitutes the population of documents for the present study. Conceived by Eugene Garfield but now maintained by Clarivate Analytics, WoS is the top scientific citation search and analytical information platform worldwide, serving both as a multidisciplinary research tool supporting a variety of scientific tasks and as a dataset for large, data-intensive studies [18].

The use of the WoS databases enables the analysis of all institutional affiliations reported in the documents and the calculation of citation indicators. The WoS brings together the most visible literature at a global level. These qualities justify its choice as the database platform used in this study despite some limitations related to covering non-English biomedical journals [18].

Although initially no limitations were imposed on our search, to calculate the bibliometric indicators we considered only two types of documents, articles and reviews, as these are the primary references for researchers. The study period was limited to 2001–2015, as delays associated with assigning MeSH descriptors to documents mean that information on the most recent articles on pneumonia is not updated. The searches took place on the Clarivate Analytics WoS platform, which includes MEDLINE database, on March 20, 2018.

### Analyzing bibliographic characteristics and standardizing data

For each of the retrieved documents, data on the following bibliographic characteristics were extracted: year of publication, journal of publication and WoS subject category, document type, authorship, citations, institutional affiliation(s), and MeSH descriptors.

Data were then standardized: institutional affiliations corresponding to England, Northern Ireland, Scotland and Wales were grouped together under “United Kingdom,” while affiliations in Overseas France, British Overseas Territories, and island dependencies were also assigned to their ruling countries (for example, the documents signed by authors from French Polynesia, Guadeloupe, Martinique, New Caledonia, and Reunion were assigned to France), although regional designations correspond to geographical rather than political criteria. Scientific production from Taiwan, which in WoS is considered independently from the Democratic Republic of China (China) but whose status is disputed at an international level, was analyzed separately.

Countries responsible for publications were categorized according to their World Bank classification by income level: low-income (< USD 1025), lower-middle-income (USD 1026 to USD 4035), upper-middle-income (USD 4036 to USD 12,475), and high-income (≥ USD 12,476) countries. Each of the countries identified was assigned to a macro geographical (continental) region according to the groups established by the World Bank based on geopolitical and economic criteria and reflected in the World Bank Country and Lending Groups (see Additional file 1: Tables S2 and S3) [19]**.**

### Calculating indicators

Two kinds of indicators were obtained:

#### Descriptive indicators for the evolution of scientific production

We analyzed the evolution of the number of documents by year of publication and according to three 5-year periods: 2001–2005, 2006–2010, and 2011–2015. Indicators also included the frequency of publication by country, geographical region, journal and MeSH descriptor; the rate of growth in scientific production from the first to the third quinquenniums, calculated as the difference between the number of publications in 2011–2015 and those from 2001 to 2005, divided by the number of publications from 2001 to 2005.

#### Production by country, adjusted for demographic and economic parameters as well as for human resources dedicated to research activities

We determined standardized indicators for each country’s productivity with respect to:

- Population: number of publications per million inhabitants (population index).
- Gross domestic product (GDP): numbers of publications per 1 billion US dollars of GPD (GPD index).
- Gross national income (GNI) per capita: number of publications per 100 US dollars of GNI per capita (GNI per capita index).
- Research and development (R&D) expenditure: numbers of publications per % of GDP expenditure in R&D (R&D expenditure index).
- Researchers in R&D: numbers of publications per researcher per million inhabitants (Researchers in R&D index)

Data were obtained from World Development Indicators in the World Bank online databases [20]. We calculated a mean value for each indicator based on available data from the study period. The analysis was limited to countries participating in the top 30 articles in the field of pneumonia in order to facilitate comparison between countries’ scientific production, demographic indicators, and economic development. Results for the top 15 articles are shown in the main text, while those for the top 30 are provided in Additional file 1.

### Citation indicators

We calculated the following citation indicators by journal, country, and geographic region:

- Citation of the publications. Absolute number of citations received.
- Citation rate (CR). Number of citations divided by number of publications.
- Hirsch index (h-index). The H-index is a semiqualitative proxy measure to assess the impact of an author’s or country’s research output on the scientific community [21]. An h-index of 12 indicates that 12 out of 12 published papers have been cited at least 12 times.

In order to assess the differences in the distributions of the publications according to the prestige of the journals, we performed a specific analysis of a sub-sample of publications in journals occupying the top 10% in the impact factor ranking in their respective subject categories in the Journal Citation Reports (2015 edition). We analyzed participation in these “prestigious journals” according to geographical location (regions and countries), collaboration level and number of citations.

### Collaboration indicators and network analysis

We calculated the percentage of documents produced in international collaboration and the evolution by quinquennium in order to estimate the scope of cooperative practices at a global level, considering the whole population of documents analyzed (research field) by country and geographic region. To specifically analyze collaboration between countries, collaboration networks were generated for each of the three quinquenniums using Pajek software. To specifically analyze collaboration between countries, collaboration networks were generated for each of the three quinquenniums using Pajek software. The collaboration network is a graphic representation (graph), wherein the nodes represent authors’ countries (as determined from their institutional affiliations) and links between the nodes represent coauthorships between countries, that is, an international collaboration in published research. The more intense the collaboration, the thicker the links between the nodes. The spatial distribution of the nodes responds to the execution of the kamada-kawai algorithm in Pajek, which places the most prominent nodes (those with a greater number of documents and collaboration links) in the center of the map, and the nodes with a smaller number of publications and degree of collaboration towards the periphery.

### Analysis of the main topics addressed in research

Based on an analysis of MeSH terms, we identified the main research focus of the studies in the area, generating density maps using the VOSviewer program with a spatial description of the main MeSH terms for each type of pneumonia [22]: (A) “Pneumonia, Aspiration” (B) “Pneumonia, Bacterial,” (C) “Pneumonia, Ventilator-Associated,” (D) “Pneumonia, Viral,” and (E) “Pneumonia, Pneumocystis”). The process of generating and interpreting the maps proceeded as follows:

- Determination of the co-occurrence of the descriptors assigned to the documents and generation of a matrix of absolute values. The joint assignment of two descriptors in a single document implies a thematic affinity, as both aspects are addressed simultaneously in the same paper. This affinity will be more intense as it is repeated a greater number of times in the collection of documents analyzed.
- Elimination of generic descriptors. In order to facilitate the analysis, we eliminated some excessively generic descriptors (like “humans” or “animals”), along with geographical descriptors and those related to age groups. These descriptors showed very high-density relationships, complicating the analysis and the interpretation of the results, so we analyzed their frequency more specifically.
- Visual representation of the network. To establish the main topics that exist for each type of pneumonia and to represent them visually, we used a clustering algorithm in the VOSViewer program, which helps to detect the communities (clusters) within a network, made up of groups of homogeneous items that are strongly related to each other. The different groupings, in the form of “islands” in red tones, represent the main clusters of the thematic networks, while the chromatic gradation illustrates the areas with a lower density of relations between the MeSH in yellow and green tones. The spatial distribution of the MeSH and their proximity to each other responds to the intensity of co-occurrence between them.

All data used to perform the study, including the information downloaded from the database as well as that derived from the treatment of the bibliographic entries, are available in the Dataverse Project, an open access public repository [23] (https://dataverse.harvard.edu/, doi: 10.7910/DVN/02BUNE).

### Ethical aspects

Due to the nature of the study and dataset, it was not necessary to obtain informed consent or approval from an institutional ethics committee.

## Results

### Evolution of scientific production and distribution by country and geographic region

The search yielded a total of 33,944 documents published between 2001 and 2015 and assigned with the descriptor “Pneumonia” in the MEDLINE database. Of these, 27,017 (79.59%) were indexed in the WoS Core Collection Databases; 20,918 (77.14%) of them were classified as articles and 1776 (6.57%) as reviews. Thus, the population of study documents was a dataset of 22,694 articles and reviews, which we used to calculate the indicators presented below. Letters (*N* = 2213; 8.19%), editorials (*N* = 1, 998; 7.39%), news (*N* = 58; 0.21%), proceedings (*N* = 17; 0.06%) and other document types (*N* = 31, 0.11%) were excluded from the analysis.

The number of publications rose from 981 in 2001 to 1977 in 2015.The evolution of scientific production by year was fitted to a linear growth model, showing an R^2^ value of 0.956. Overall, the study period saw a two-fold increase in scientific production (Additional file 1: Figure S1).

The country with the greatest number of documents was the USA (38.49%), followed at some distance by the UK (7.18%), Japan (6.97%), Germany (6.80%) and France (6.73%). Table 1 shows the number of documents and the evolution of scientific production in the 15 most productive countries by quinquennium (see Additional file 1: Table S4 for results on the top 30 countries).

**Table 1:** Top 15 countries ranked by total number of publications by quinquenniums 2001–2005, 2006–2010, and 2010–2015

Total				2001–2005			2006–2010			2011–2015		
Country	N of docs	%	^a^ PPD	Country	N of docs	%	Country	N of docs	%	Country	N of docs	%
USA	8735	38.49	−4.61	USA	2248	41.13	USA	2907	39.14	USA	3580	36.52
UK	1629	7.18	0.81	France	417	7.63	Germany	521	7.01	China	827	8.44
Japan	1581	6.97	0.03	UK	403	7.37	Japan	518	6.97	Japan	725	7.40
Germany	1544	6.80	0.18	Germany	388	7.10	UK	512	6.89	UK	714	7.28
France	1527	6.73	0.30	Japan	338	6.18	France	498	6.71	Germany	635	6.48
Spain	1251	5.51	0.81	Spain	297	5.43	Spain	423	5.70	France	612	6.24
China	1126	4.96	0.11	Canada	290	5.31	Canada	361	4.86	Spain	531	5.42
Canada	1091	4.81	0.74	Netherlands	205	3.75	Italy	298	4.01	Canada	440	4.49
Netherlands	911	4.01	1.43	Italy	160	2.93	Netherlands	279	3.76	Netherlands	427	4.36
Italy	859	3.79	1.35	Australia	150	2.74	China	237	3.19	Italy	401	4.09
Australia	734	3.23	1.32	Switzerland	128	2.34	Australia	225	3.03	Australia	359	3.66
Brazil	600	2.64	1.62	Belgium	87	1.59	Brazil	213	2.87	South Korea	315	3.21
Switzerland	541	2.38	1.65	Sweden	84	1.54	Switzerland	190	2.56	Brazil	313	3.19
South Korea	534	2.35	1.5	Denmark	83	1.52	Taiwan	149	2.01	Taiwan	296	3.02
Taiwan	509	2.24	0.76	Turkey	83	1.52	South Korea	148	1.99	Switzerland	223	2.28

*N of docs = numbers of documents*

^*a*^ *PPD = Percentage point difference from 2001 to 2005 to 2011–2015*

Although the USA ranks first in all periods, its relative contributions have declined, from 41.13% of all documents in 2001–2005 to 36.52% in 2011–2015. On the other hand, China’s emergence is highly notable, with a 1.13% share of total scientific production in the first period (rank = 22), compared to a 8.44% share in the third (rank = 2). South Korea has also seen considerable growth, contributing just 1.30% to total research production in 2001–2005 (rank = 19) but 3.21% in 2011–2015 (rank = 12). Likewise, Taiwan and Brazil have increased their production from 1.17 and 1.35%, respectively, to 3.02 and 3.19%.

Scientific production in different countries and geographic regions, and its evolution by quinquennium, is concentrated in North America and Europe & Central Asia; together these regions are responsible for 82.87% of the papers included in the population of documents. Research in the two regions has decreased the proportion of documents from 2001 to 2005 to 2011–2015 (− 5.46 and − 4.56%). Countries from East Asia & the Pacific and from Latin America & the Caribbean contributed with 20.90 and 4.84% of the documents, respectively. Growth was pronounced in these regions, at 13.18 and 2.52%. Table 2) (see Additional file 1: Figure S2 for a visual representation of density equalizing mapping projections).

**Table 2:** Geographical regions and income brackets by total number of publications and quinquennium 2001–2005, 2006–2010, and 2010–2015

	Total			2001–2005		2006–2010		2011–2015	
	N of docs	%	^a^ PPD	N of docs	%	N of docs	%	N of docs	%
Geographic area									
North America	9549	42.08	−5,46	2469	45.18	3187	42.91	3893	39.72
Europe & Central Asia	9256	40.79	−4,54	2359	43.17	3110	41.87	3787	38.63
East Asia & Pacific	4742	20.90	13,18	743	13.60	1374	18.50	2625	26.78
Latin America & Caribbean	1099	4.84	2,52	174	3.18	366	4.93	559	5.70
Middle East & North Africa	590	2.60	0,93	115	2.10	178	2.40	297	3.03
Sub-Saharan Africa	523	2.30	0,35	121	2.21	151	2.03	251	2.56
South Asia	461	2.03	1,48	56	1.02	160	2.15	245	2.50
Income bracket			0						
HI	20,102	88.58	−7,76	5092	93.17	6638	89.38	8372	85.41
UMI	3094	13.63	10	434	7.94	902	12.14	1758	17.94
LMI	803	3.54	2,43	109	1.99	261	3.51	433	4.42
LI	222	0.98	0,74	32	0.59	60	0.81	130	1.33

*N of docs = numbers of documents*

^*a*^ *PPD = Percentage point difference from 2001 to 2005 to 2011–2015*

*HI high-income, UMI upper-middle-income, LMI = lower-middle-income, LI = low-income*

### Number of publications by country relative to population and economic parameters

Table 3 ranks the production of the top 15 countries, adjusted for demographic and economic indicators (see Additional file 1: Table S5 for results on the top 30 countries). When normalized by population, the most productive countries were Switzerland, the Netherlands, Iceland, and Denmark. Adjusted for the GDP index, the most productive LMICs were the Gambia, Malawi, Uganda, and Guinea Bissau. If we calculate the ratio of pneumonia publications to GNI per capita index, the USA, China, India, Malawi y Brazil were the most productive. Adjusting by R&D expenditure index, the USA ranked first, followed by Spain, the UK, China, and Italy. In relation to the researchers in R&D index, the USA also leads the ranking, followed by India, Uganda, and China. (see Additional file 1: Figure S3 and Figure S4 for a visual representation of density equalizing mapping projections of the number of documents and world development indicators, by GNI per capita index, GDP index and population index plus R&D expenditure index).

**Table 3:** Top 15 countries and world regions ranked according to population index, GDP index, GNI per capita index, R&D expenditure index and Researchers in R&D Index^b,a^

Country^a^	Population Index^b^	Country	GPD Index^c^	Country	GNI per capita Index^d^	Country	R&D expenditure Index^e^	Country	Researchers in R&D Index^f^
Switzerland	70.32	Gambia	30.83	USA	18.31	USA	3276.91	USA	2.25
Netherlands	55.23	Malawi	9.27	China	14.08	Spain	1056.90	India	1.84
Iceland	51.70	Uganda	3.42	India	8.25	UK	993.78	Uganda	1.39
Denmark	50.54	Guinea Bissau	2.62	Malawi	5.19	China	735.50	China	1.22
Finland	40.77	Andorra	1.94	Brazil	4.83	Italy	731.10	Malawi	1.16
Belgium	37.29	Kenya	1.88	UK	4.67	France	712.03	Brazil	1.06
Sweden	35.94	Vanuatu	1.78	Japan	4.54	Germany	589.28	Tanzania	0.78
Israel	35.05	Cambodia	1.60	France	4.40	Canada	579.61	Cambodia	0.67
Australia	34.24	Nepal	1.55	Spain	4.20	Brazil	557.01	South Africa	0.62
Canada	32.71	Grenada	1.35	Germany	4.06	Turkey	532.13	Italy	0.54
USA	28.78	Israel	1.26	Uganda	4.04	Netherlands	500.78	Philippines	0.53
Spain	27.90	Papua N Guinea	1.26	Bangladesh	3.07	Japan	493.90	Colombia	0.52
Greece	26.84	Mozambique	1.25	Canada	2.89	Greece	448.47	Mozambique	0.52
UK	26.32	Netherlands	1.22	Kenya	2.86	Thailand	445.07	Turkey	0.51
New Zealand	25.65	Tunisia	1.19	Italy	2.59	Gambia	423.33	Ghana	0.50

^*a*^ *Monaco has a population index of 112.42 and Andorra, 75.86; these countries were excluded due to their especially small size and population* ^b^ *Number of publications per million inhabitants*

^c^ *Number of publications per 1 billon US dollars of gross domestic product (GPD)*

^*d*^*Number of publications per 100 USD dollars of gross national income (GNI)* per capita

^*e*^ *Numbers of publications per % of GDP expenditure in Research and Development (R&D)*

^*f*^ *Numbers of publications per researcher per million inhabitants*

### Impact of publications

The citation analysis by geographical regions reflects the balance in the absolute number of citations received by researchers in North America and Europe, with the rest of the regions trailing considerably. In contrast, North America presents a somewhat higher citation rate (CR) than Europe (35.76 versus 29.20); among the other regions, Africa showed the best performance on this indicator (CR 31.41), with the rest presenting values of 20.07 to 24.00. In consonance with these data, at a country level the HICs (which are mostly in Europe and North America) showed higher CRs than countries in the rest of the income categories. By individual country, articles with author affiliations from the USA were the most cited (*N* = 316,942), followed by articles from the UK (*N* = 62,612), France (*N* = 48,019), Spain (*N* = 43,459) and Germany (N = 43,434). Regarding the country-specific CR, Vietnam dominated (CR 50.79), followed by the Switzerland (CR 42.94), South Africa (CR 42.85), New Zealand (CR 40.49), Saudi Arabia (CR 38.62) and the UK (CR 38.44). The USA and the UK were the top-ranked countries with an h-Index of 197 (USA) and 106 (UK), followed by France (96), Spain (94) and Germany (94) (Table 4) (see Additional file 1: Table S6 for the 30 most productive countries).

**Table 4:** Citation indicators for pneumonia research: rankings by 15 top-producing countries, geographic region and income (2001–2015)

	Citations		Citation Rate		H-index
Country					
USA	316,942	Vietnam	50.79	USA	197
UK	62,612	Switzerland	42.94	UK	106
France	48,019	South Africa	42.85	France	96
Spain	43,459	New Zealand	40.49	Spain	96
Germany	43,436	Saudi Arabia	38.62	Germany	94
Canada	40,090	UK	38.44	Canada	88
Netherlands	34,798	Netherlands	38.20	Netherlands	88
Japan	30,978	Ireland	36.85	Japan	74
Italy	25,600	Canada	36.75	Switzerland	74
Switzerland	23,228	Sweden	36.65	Australia	71
Australia	22,440	Denmark	36.53	Italy	70
China	18,370	USA	36.28	Belgium	62
Belgium	13,919	Spain	34.74	Sweden	56
Sweden	12,203	Belgium	34.71	Denmark	55
Brazil	11,136	Finland	34.17	China	54
Geographic area					
North America	341,438	North America	35.76	North America	202
Europe & Central Asia	270,237	Europe & Central Asia	29.20	Europe & Central Asia	172
East Asia & Pacific	96,628	Sub-Saharan Africa	31.41	East Asia & Pacific	103
Latin America & Caribbean	22,740	Middle East & North Africa	24.00	Latin America & Caribbean	61
Sub-Saharan Africa	16,426	Latin America & Caribbean	20.69	Sub-Saharan Africa	54
Middle East & North Africa	14,159	East Asia & Pacific	20.38	Middle East & North Africa	53
South Asia	9254	South Asia	20.07	South Asia	46
Countries by income					
HIC	593,632	HIC	29.53	HIC	222
UMIC	58,785	LMIC	21.82	UMIC	89
LMIC	17,523	LIC	21.46	LMIC	60
LIC	4765	UMIC	19.00	LIC	34

*HIC high-income countries, UMIC upper-middle-income countries, LMIC lower-middle-income countries, LIC low-income countries*

### Analysis of international collaboration

Overall, 18.80% of the articles published in the study period were written in international collaboration, although the rates increased from 14.35% in the 2001–2005 quinquennium to 21.64% in 2011–2015. Among the top 15 most productive countries, international collaboration was much more intense in the European countries, Brazil, Canada, and Australia (34 to 62%) compared to the USA (26.33%) and the most productive countries of East Asia & Pacific (China, South Korea, and Taiwan: 16 to 28%) (Table 5). The very high levels of international collaboration are even more pronounced in some Latin American, South Asia and particularly African countries. Indeed, the analysis of collaboration by geographical regions shows that globally, sub-Saharan Africa collaborated on 46.08% of the papers produced. The results for Latin America and the Caribbean (22.66%) are heavily weighted by research from Brazil, but the rates of international collaboration were 63.01% in Colombia, 60.94% in Argentina, and 52.21% in Mexico, while in East Asia & Pacific and South Asia (and looking beyond the most productive countries like China), countries like Bangladesh showed levels of international collaboration of 73.61%; Thailand, 60.29%; and Pakistan, 58.82%.

**Table 5:** Rates of international collaboration (%) in the top 15 most productive countries and by world region, pneumonia research output (2001–2015)

	Total			2001–2005			2006–2010			2011–2015		
	N docs	N docs Int col	%	N docs	N docs Int col	%	N docs	N docs Int col	%	N docs	N docs Int col	%
Country												
USA	8735	2300	26.33	2248	427	18.99	2907	761	26.18	3580	1112	31.06
UK	1629	811	49.82	403	156	38.71	512	241	47.07	714	414	57.98
Japan	1581	285	18.03	338	58	17.16	518	94	18.15	725	133	18.34
Germany	1544	626	40.54	388	113	29.12	521	186	35.70	635	327	51.50
France	1527	513	33.59	417	98	23.50	497	155	31.19	613	260	42.41
Spain	1251	422	33.73	297	61	20.54	423	124	29.31	531	237	44.63
Peoples R. China	1126	320	28.42	62	21	33.87	237	91	38.40	827	208	25.15
Canada	1091	503	46.10	290	112	38.62	361	145	40.17	440	246	55.91
Netherlands	911	414	45.44	205	69	33.66	279	127	45.52	427	218	51.05
Italy	859	345	40.16	160	43	26.88	298	115	38.59	401	187	46.63
Australia	734	355	48.37	150	65	43.33	225	111	49.33	359	179	49.86
Brazil	600	216	36	74	30	40.54	213	75	35.21	313	111	35.46
Switzerland	541	337	62.29	128	62	48.44	190	123	64.74	223	152	68.16
South Korea	534	105	19.66	71	19	26.76	148	32	21.62	315	54	17.14
Taiwan	509	83	16.31	64	11	17.19	149	23	15.44	296	49	16.55
Total international collaboration	22,593	4248	18.80	5442	781	14.35	7373	1351	18.32	9778	2116	21.64
Geographic area												
North America	9549	1276	13.36	2469	216	8.75	3187	407	12.77	3893	653	16.77
Europe & Central Asia	9256	1033	11.16	2359	167	7.08	3110	341	10.96	3787	525	13.86
East Asia & Pacific	4742	610	12.86	743	100	13.46	1374	209	15.21	2625	301	11.47
Latin America & Caribbean	1099	249	22.66	174	45	25.86	366	68	18.58	559	136	24.33
Middle East & North Africa	590	110	18.64	115	14	12.17	178	28	15.73	297	68	22.90
Sub-Saharan Africa	523	241	46.08	121	43	35.54	151	67	44.37	251	131	52.19
South Asia	461	108	23.43	56	10	17.86	160	33	20.63	245	65	26.53
Total world region collaboration	22,593	3109	13.76	5442	536	9.85	7373	1007	13.66	9778	1566	16.02

**Fig. 1:**
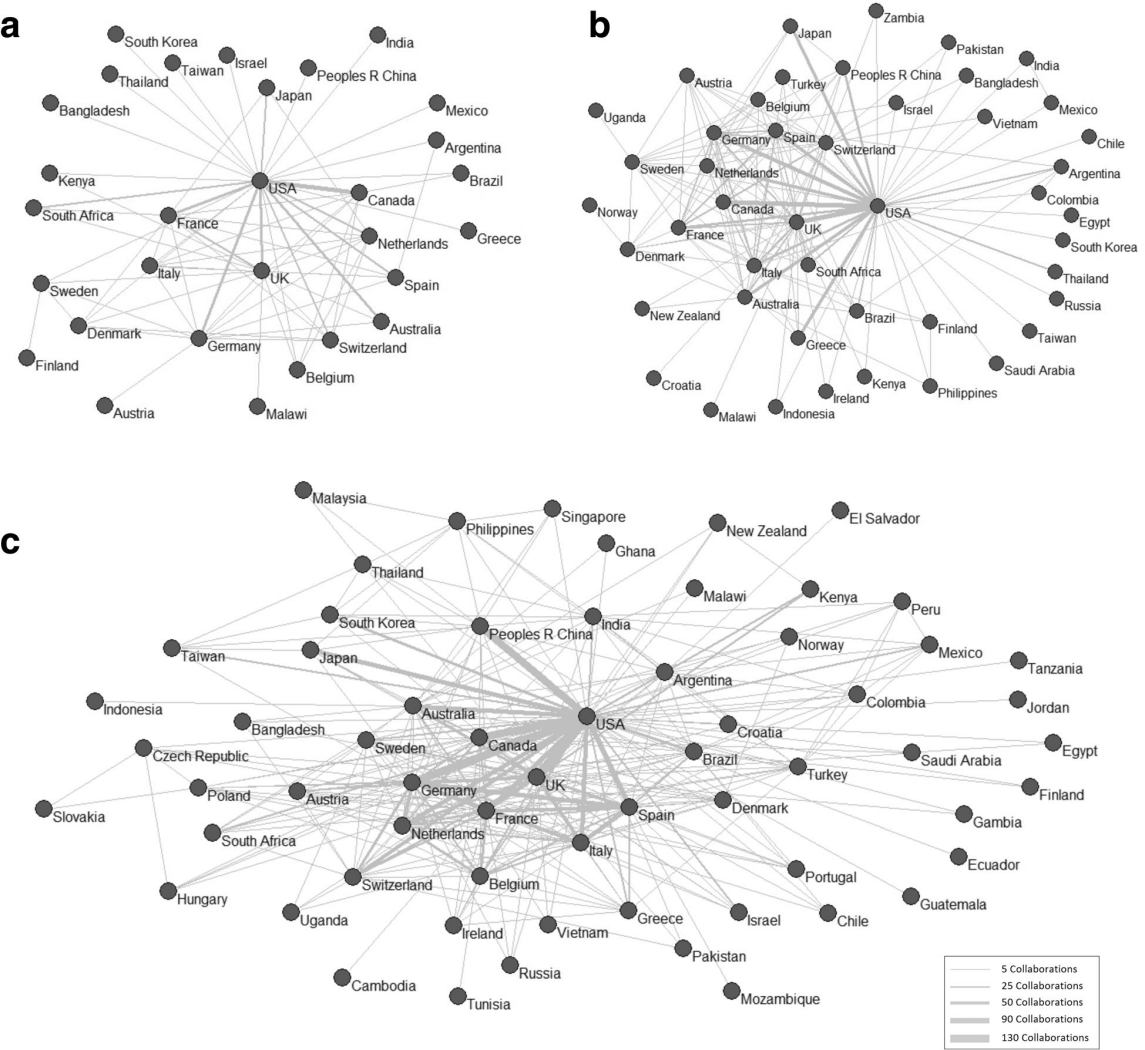
Networks generated from international collaborations, by quinquennium: (**a**) 2001–2005, (**b**) 2006–2010, and (**c**) 2011–2015 The intensity of collaboration is reflected through the thickness of the links. The most prominent nodes (those with a greater number of documents and collaboration links) are in the center of the map, while the nodes with a smaller number of publications and lower degree of collaboration are located on the periphery

Figure 1 shows the collaboration networks between different countries by quinquennium. The most prominent countries in all time periods, occupying central positions in the networks with multiple cooperative links, are the USA, Canada, the UK, Germany, France, and the Netherlands. The presence of South American and African countries is scarce in all periods. Only South Africa has a notable presence in the third quinquennium (Fig. 1a). A few other countries also “emerge” with a high degree of collaborative links in the second period, like Spain, Greece, Italy, Australia, China, and Japan, although the latter two countries are not fully integrated in global networks, showing collaborative ties only with the USA (Fig. 1b). Finally, other European countries, while present throughout all three periods, stand out to a greater degree in the third period. This is the case of Sweden, Switzerland, Belgium, and Austria. At the same time, China and Japan seem more implicated in the network in this third period, while India and South Korea also gain relevance (Fig. 1c).

### Journals of publication

The documents we analyzed were published in 2115 scientific journals. Twelve journals accounted for 16.63% of the pneumonia literature Table 6

**Table 6:** Top 15 most productive journals and their citation indicatiors, pneumonia research 2001–2015)

Top 15 journals	N. of docs	%	CR	Impact factor 2015	Journal category (ranking)
*PLOS ONE*	494	2.18	15.12	3.057	Multidisciplinary Sciences (11 of 63)
*Clinical Infectious Diseases*	412	1.81	63.96	8.736	Immunology (9 of 151) Infectious Diseases (2 of 83) Microbiology (10 of 123)
*Chest*	397	1.75	55.95	6.136	Respiratory System (6 of 58) Critical Care Medicine (5 of 33)
*Journal of Immunology*	354	1.56	49.10	4.985	Immunology (32 of 151)
*American Journal of Physiology-Lung Cellular and Molecular Physiology*	323	1.42	34.96	4.721	Physiology (8 of 83) Respiratory System (8 of 58)
*Critical Care Medicine*	291	1.28	55.15	7.422	Critical Care Medicine (4 of 33)
*European Respiratory Journal*	283	1.25	42.49	8.332	Respiratory System (3 of 58)
*Infection and Immunity*	256	1.13	37.77	3.603	Immunology (56 of 151) Infectious Diseases (20 of 83)
*American Journal of Respiratory And Critical Care Medicine*	256	1.13	88.46	13.118	Critical Care Medicine (2 of 33) Respiratory System (2 of 58)
*American Journal of Respiratory Cell and Molecular Biology*	251	1.11	32.77	4.082	Biochemistry & Molecular Biology (74 of 289) Cell Biology (64 of 187) Respiratory System (10 of 58)
*Antimicrobial Agents and Chemotherapy*	213	0.94	27.84	4.415	Microbiology (22 of 123) Pharmacology & Pharmacy (34 of 255)
*Intensive Care Medicine*	212	0.93	42.65	10.125	Critical Care Medicine (3 of 33)
*Journal of Clinical Microbiology*	209	0.92	29.54	3.631	Microbiology (36 of 123)
*Pediatric Infectious Disease Journal*	196	0.86	28.09	2.587	Immunology (84 of 151) Infectious Diseases (38 of 83) Pediatrics (22 of 120)
*Vaccine*	190	0.84	22.98	3.413	Immunology (60 of 151) Medicine. Research & Experimental (36 of 124)

*CR citation rate*

. shows a list of the 15 top journals with the highest number of papers published from 2001 to 2015, as well as their impact factors for the year 2015, subject category according to the Journal Citation Reports classification, and CR (Additional file 1: Table S7 for results on the top 30 journals). The journals publishing the most articles on pneumonia were *PLOS ONE* (*N* = 494)*, Clinical Infectious Diseases* (*N* = 412), and *Chest* (*N* = 397), whereas the journals with the most citations were *Clinical Infectious Diseases*, (*N* = 26,351), *American Journal of Respiratory and Critical Care* (*N* = 22,647), and *Chest* (N = 22,212); all of these were also among the 15 most productive journals. The journals with the highest CRs were the *New England Journal of Medicine* (75 documents, CR 278.13)*, The Lancet* (54 documents, CR 210.17) and *JAMA* (49 documents, CR = 199.71) (see Additional file 1: Table S8 for results on the top 30 journals with highest absolute and relative citations).

The comparative analysis of the scientific production and CRs of different journals is noteworthy in that some journals (such as the *American Journal of Respiratory and Critical Care*, *Critical Care Medicine*, and *Intensive Care Medicine*) present a very high CR in relation to their total scientific production (Additional file 1: Figure S5 for the top 15 journals producing the most research on pneumonia, plus citation rates).

With regard to the subject categories to which the journals are assigned, the most prominent are “Infectious Diseases” (17.57% of the documents), “Respiratory System” (15.77%), “Immunology” (14.08%), “Microbiology” (11.85%), and “Critical Care Medicine” (9.26%) Table 7.

**Table 7:** Top 15 Web of Science Categories in pneumonia research (2001–2015)

	2001–2015		2001–2005		2006–2010		2011–2015	
WoS Category	N	%	N	%	N	%	N	%
Infectious Diseases	3987	17.57	957	17.51	1374	18.50	1656	16.89
Respiratory System	3579	15.77	989	18.10	1192	16.05	1398	14.26
Immunology	3195	14.08	799	14.62	1143	15.39	1253	12.78
Microbiology	2690	11.85	725	13.27	899	12.10	1066	10.88
Critical Care Medicine	2101	9.26	584	10.69	742	9.99	775	7.91
Medicine, General & Internal	2038	8.98	569	10.41	622	8.37	847	8.64
Pharmacology & Pharmacy	1664	7.33	382	6.99	526	7.08	756	7.71
Pediatrics	1574	6.94	437	8.00	565	7.61	572	5.84
Surgery	1091	4.81	270	4.94	387	5.21	434	4.43
Public, Environmental & Occupational Health	962	4.24	187	3.42	330	4.44	445	4.54
Veterinary Sciences	879	3.87	273	5.00	268	3.61	338	3.45
Medicine, Research & Experimental	714	3.15	149	2.73	223	3.00	342	3.49
Biochemistry & Molecular Biology	661	2.91	143	2.62	194	2.61	324	3.31
Cell Biology	602	2.65	150	2.74	170	2.29	282	2.88
Multidisciplinary Sciences	576	2.54	7	0.13	65	0.88	504	5.14

Many of the most productive journals in pneumonia also fall into these subject categories. Moreover, over the course of the three study periods, nearly all of the subject categories saw a moderate decrease in their relative contribution, as research articles became more dispersed and made headway into different disciplines producing less research on pneumonia Table 7.

**Table 8:** Distribution of participation by countries in the most prestigious 10% of journals

Country	N of docs	%	Rank	N docs International collaboration	%	N cites	Citation Rate	Rank
USA	1954	47.66	1	627	32.09	139,247	71.26	1
UK	473	11.54	2	263	55.6	34,471	72.88	2
Japan	132	3.22	11	55	41.67	6782	51.38	11
Germany	285	6.95	5	177	62.1	16,636	58.37	7
France	401	9.78	3	152	37.9	26,174	65.27	3
Spain	373	9.1	4	173	46.38	25,387	68.06	4
China	105	2.56	12	51	48.57	4926	46.91	14
Canada	271	6.61	6	141	52.03	19,291	71.18	5
Netherlands	256	6.24	7	118	46.09	16,820	65.7	6
Italy	174	4.24	8	111	63.79	11,626	66.82	9
Australia	161	3.93	9	89	55.28	9688	60.17	10
Brazil	78	1.9	14	49	62.82	2629	33.7	22
Switzerland	154	3.76	10	113	73.38	13,206	85.75	8
South Korea	50	1.22	19	19	38	2226	44.52	23
Taiwan	41	1	22	15	36.58	1568	38.24	30

### Analysis of collaboration and citation in a top 10% de las prestigious journals

The analysis of the 4100 documents published in the top 10% of prestigious journals shows a higher participation from the USA (27.66%, compared to 38.49% in the overall body of documents) and from some other European countries like the UK or Spain. In contrast, the weight of Asian countries, particularly Japan and China, is much lower (Table 8). Overall, international collaboration in these journals (*N* = 1065, 25.98%) was sensibly higher than in the overall body of documents (18.8%), and the greater degree of collaboration was much more pronounced for countries like Brazil, Japan, China, and even European countries like Italy and Germany (Table 8).

The high degree of collaboration was also confirmed between regions in the publications appearing in these journals (Table 9). With regard to the degree of citation, we observed notable increases in the citation rate of the USA and the European countries; these were even more significant for countries in the Middle East & North Africa, and for sub-Saharan Africa when they participated in these journals (Table 9).

**Table 9:** Distribution of participation by countries in the most prestigious 10% of journals

Geographic area	N of docs	%	*N docs world region collaboration*	%	Citation	Citation Rate
North America	2138	52.15	630	29.47	149,290	69.83
Europe & Central Asia	1978	48.24	600	30.33	125,727	63.56
East Asia & Pacific	543	13.24	241	44.38	28,248	52.02
Latin America & Caribbean	152	3.71	109	71.71	8246	54.25
Middle East & North Africa	75	1.83	45	60	6383	85.11
Sub-Saharan Africa	105	2.56	93	88.57	8568	81.6
South Asia	70	1.71	51	72.86	3855	55.07

### Analysis of subject areas; frequency and distribution of MeSH terms

With regard to types of pneumonia studied, the MeSH terms to appear most frequently were “Pneumonia, Bacterial” (19.99%), followed by “Pneumonia, Pneumococcal” (7.02%), and “Pneumonia, Ventilator-Associated” (6.79%). Table 10 shows the number of documents assigned to each term describing the different types of pneumonia (Additional file 1: Table S10 for the 30 top general MeSH).

**Table 10:** Number of documents assigned to MeSH terms describing different types of pneumonia

MeSH Term	N of docs	%
Pneumonia MeSH		
Pneumonia, Bacterial	4536	19.99
Pneumonia, Pneumococcal	1593	7.02
Pneumonia, Ventilator-Associated	1542	6.79
Pneumonia, Pneumocystis	1323	5.83
Pneumonia, Viral	1212	5.34
Pneumonia, Aspiration	1109	4.89
Pneumonia, Mycoplasma	887	3.91
Pneumonia, Staphylococcal	423	1.86
Bronchopneumonia	310	1.37
Pneumonia of Swine, Mycoplasmal	226	1.00
Pleuropneumonia	129	0,57
Pneumonia, Lipid	70	0.31
Pneumonia of Calves, Enzootic	38	0.17
Chlamydial Pneumonia	24	0.11
Pneumonia, Rickettsial	2	0.01
Pneumonia, Necrotizing	0	0.00

*N of docs numbers of documents*

Table 11 ranks the top 15 countries in crude numbers of retrieved articles, stratified by types of pneumonia (Additional file 1: Table S11 for information on the 30 most productive countries). For “Pneumonia, Aspiration”, the main countries were the USA, Japan, and Germany; for “Pneumonia, Bacterial”, the USA, France, and Spain; for “Pneumonia, Pneumocystis”, the USA, France, and the UK; for “Pneumonia, Ventilator-Associated”, the USA, France, and Spain; and for “Pneumonia, Viral”, the USA, China, and Japan.

**Table 11:** Distribution of research articles on different pneumonia types amont 15 most productive countries

Pneumonia, Aspiration		Pneumonia, Bacterial		Pneumonia, Pneumocystis		Pneumonia, Ventilator-Associated		Pneumonia, Viral	
Country	N of docs	Country	N of docs	Country	N of docs	Country	N of docs	Country	N of docs
USA	394	USA	1709	USA	525	USA	650	USA	383
Japan	169	France	379	France	149	France	170	China	98
Germany	78	Spain	378	UK	106	Spain	139	Japan	95
UK	74	Germany	329	Japan	104	Greece	72	UK	83
Australia	45	Japan	297	Spain	64	Canada	69	Germany	81
Canada	44	UK	252	Germany	58	UK	68	Spain	71
France	40	Canada	209	Italy	46	Germany	67	France	66
Spain	39	Italy	176	Switzerland	38	China	63	Italy	59
Turkey	31	Netherlands	173	China	38	Brazil	63	Canada	48
China	25	China	171	South Africa	35	Italy	63	Netherlands	47
Italy	24	Australia	123	Denmark	28	Turkey	58	South Korea	41
South Korea	22	Taiwan	104	Canada	27	Netherlands	53	Finland	39
Switzerland	21	Switzerland	103	Taiwan	27	Australia	49	Australia	29
Netherlands	21	Brazil	100	Netherlands	25	Belgium	45	Brazil	26
Taiwan	21	South Korea	92	Australia	23	India	39	Thailand	21

*N of docs numbers of documents*

Table 12 shows the relationship between MeSH terms referring to age groups with those corresponding to different types of pneumonia. The closest associations for “Aged, 80 and over” and “Aged” were with “Pneumonia, Aspiration” (22.58 and 40.56%, respectively), while “Pneumonia, Viral” was the most frequent topic for studies in pre-adults (“Infant”, “Child”, “Child, preschool” and “Adolescent”). The one exception to this was “Infant, newborn”, where the highest proportion of articles was about “Pneumonia, Pneumocystis.” In “Adult” and “Middle aged” people, studies most frequently focused on “Pneumonia, Bacterial” and “Pneumonia, Ventilator-Associated.”

**Table 12:** Distribution of MeSH terms referring to age groups, by main types of pneumonia studied in those groups

MeSH age	Pneumonia, Aspiration			Pneumonia, Bacterial			Pneumonia, Ventilator-Associated			Pneumonia, Pneumocystis			Pneumonia, Viral		
	N of docs	rank	%	N of docs	rank	%	N of docs	rank	%	N of docs	rank	%	N of docs	rank	%
Infant, newborn	51	9	4.61	143	10	3.15	80	10	5.20	112	10	9.24	35	10	2.65
Infant	98	8	8.85	140	5	10.58	89	8	5.79	278	4	22.94	278	4	22.94
Child, preschool	100	7	9.03	91	8	6.88	85	9	5.53	268	5	22.11	268	5	22.11
Child	117	5	10.57	124	6	9.37	100	7	6.50	222	7	18.32	222	7	18.32
Adolescent	107	6	9.67	148	4	11.19	145	5	9.43	250	6	20.63	250	6	20.63
Adult	280	3	25.29	548	1	41.42	493	3	32.05	397	1	32.76	397	1	32.76
Young adult	44	10	3.97	266	9	5.86	133	6	8.65	126	9	10.40	95	7	7.18
Middle aged	366	2	33.06	502	2	37.94	680	1	44.21	348	2	28.71	348	2	28.71
Aged	449	1	40.56	288	3	21.77	496	2	32.25	281	3	23.18	281	3	23.18
Aged, 80 and over	250	4	22.58	88	9	6.65	188	4	12.22	134	8	11.06	134	8	11.06

*N of docs numbers of documents*

**Fig. 2:**
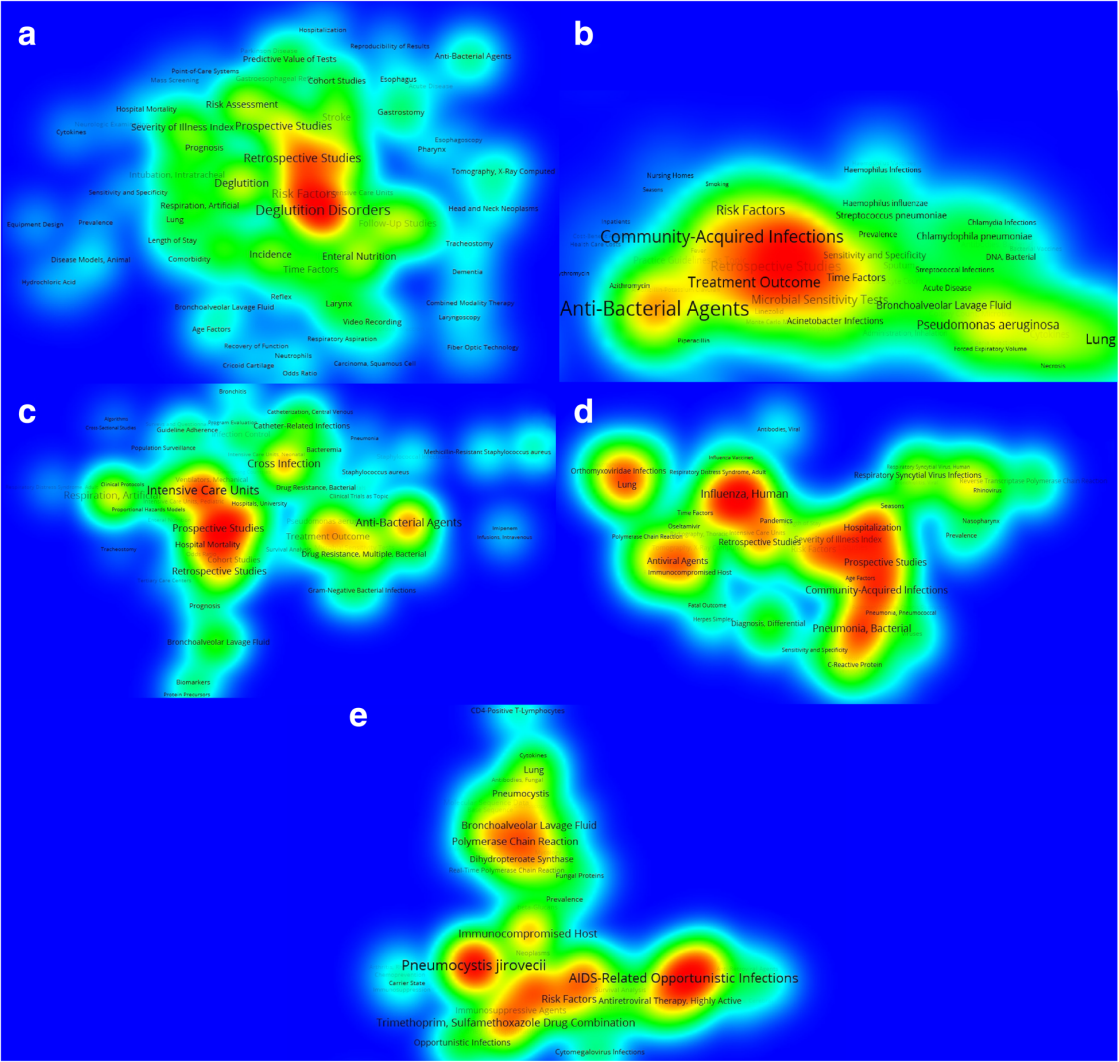
Subject area maps with the main MeSH terms associated with different types of pneumonia-(**a**) “Pneumonia, Aspiration” (**b**) “Pneumonia, Bacterial, ” (**c**) “Pneumonia, Ventilator-Associated, ” (**d**) “Pneumonia, Viral, ” and (**e**) “Pneumonia, Pneumocystis” Groupings in the form of “islands” in red tones represent the main clusters of the thematic networks, while the chromatic gradation in yellow and green tones illustrates the areas with a lower density of relations between the MeSH. The spatial distribution of the MeSH and their proximity to each other responds to the intensity of co-occurrence between them

Figure 2 shows the subject area maps with the main MeSH terms in the documents on (a) “Pneumonia, Aspiration”; (b) “Pneumonia, Bacterial”; (c) “Pneumonia, Ventilator-Associated”; (d) “Pneumonia, Viral”; and (e) “Pneumonia, Pneumocystis.” The principal MeSH term related to “Pneumonia, Aspiration” is “Deglutition Disorder”, but research is linked to a broad array of topics, including epidemiological aspects (“Incidence”, “Risk Factor”, “Retrospective Studies”), treatment approaches in intensive care, and surgical techniques procedures facilitating breathing, swallowing, and feeding (Fig. 2a).

The two main MeSH terms that appear most frequently with “Pneumonia, Bacterial” are “Community-acquired Infections” and “Anti-bacterial Agents”, reflecting the central focus that research has taken to identify risk factors and test different therapeutic approaches. MeSH terms related to specific bacteria and infections, such as *Streptococcus*, *Chlamydia*, *Acinetobacter*, and *Haemophilus influenzae*, are also prominent (Fig. 2b).

For its part, research on “Pneumonia, Ventilator-associated” seems more disperse, although three areas of interest can clearly be differentiated: (a) epidemiological studies, clinical protocols, and treatment in intensive care units (the term “Intensive Care Unit” is the most prominent in this area); (b) treatment outcomes (“Treatment outcome” and “Anti-Bacterial Agents”); and (c) cross infections (“Cross infection”) (Fig. 2c).

Research on “Pneumonia, Viral” also shows a disperse nature, with different areas of interest. Epidemiological aspects are covered under terms such as “Community-acquired Infections” and “Hospitalization”, while at a researcher level, interests reside in the virus “Influenza, Human” and “Orthomyxoviridae Infections” (Fig. 2d). With regard to “Pneumonia, Pneumocystis”, one prominent subject focus is on “AIDS-Related Opportunistic Infections” and another is on “*Pneumocystis jirovecii*” (Fig. 2e).

## Discussion

Our analysis shows that the number of publications on pneumonia increased notably over the study period, with annual research outputs doubling from 2001 to 2015. Different factors may have contributed to this. The first of these is the growing research relevance of pneumonia as a clinical entity, as this disease is one of the community-acquired infections with the highest incidence and is an important cause of hospital admissions. It is also associated with a high global burden of morbidity and mortality in both children and adults [1–3, 24]. The second potential factor relates to advances in basic immunological and microbiological research along with deepening knowledge on the pathogenesis of the disease with regard to aspects like microbiological resistance and preventive interventions (e.g. vaccines) [25]. Thirdly, increased funding has been directed toward research and particularly “proactive investments for emerging infectious threats” [8, 9], and finally, the increase in scientific production could be related to scientific development and international dissemination of scientific research in the WoS databases. This is particularly the case of China and other emerging economies like Brazil, where the rates of growth were highest relative to their respective regions [26–28].

We observed a substantial increase in research worldwide, but particularly in some geographical regions and countries of South Asia, East Asia & the Pacific, Latin America & and the Caribbean, and sub-Saharan Africa. To a great extent, this increase is simply a reflection of the limited contribution to global research that these countries made in the first period analyzed (2001–2005). The bulk of scientific production continues to come from countries with more economic and scientific development in Europe and North America (together, these countries participated in 77% of all publications).

Despite the striking increase in scientific production across LMICs, the relative contribution to pneumonia research remains very modest, and the fact that some countries rank highest in demographic and economic indicators may not be a positive feature, but rather a reflection of the scant development in their scientific systems. Furthermore, the increase in international collaboration could have played a role in these indicators, multiplying the assignment of articles to different countries and possibly inflating some values, masking the real contribution of countries with less scientific development in research activities [29].

The USA is undoubtedly the main reference for pneumonia researchers in quantitative terms, as it produces by far the largest volume of publications—four times that of the next most productive country in the last period. Other European countries with important scientific systems (e.g. the UK, Germany, France, and Spain), along with other countries like Japan, Canada, China, India, and Brazil, also stand out in relation to some of the indicators of scientific production and economic development (GNI per capita index, and R&D expenditure Index). The other significant aspect in the analysis of how scientific production evolved over the study period is the emergence of China, which in the last period of study (2011–2015) trailed only the USA in research output. This growth has come about in large part from the investments and scientific policies to foster openness that have been implemented over the past several decades to promote internationalization [30, 31].

The level of international scientific collaboration that we have observed in the field of pneumonia (19%) is below that seen in other areas of knowledge [11, 29, 30, 32–35]. Thus, even though the trend is toward increased international cooperation, rising from 14 to 22% over the study period, implementing new strategies that favor collaboration is still necessary [11].

Initiatives promoting research could include those launched by international organizations, such as the World Health Organization (WHO) and the Bill & Melinda Gates Foundation, which have both invested considerable resources to investigate the etiology of childhood pneumonia in low-income countries [36–38]. However, these initiatives carry risks too, as major actors in LMIC research, including the Bill & Melinda Gates Foundation, have been shown to be biased toward research done by researchers from HIC (doing research in LMIC) [39].

The European and Developing Countries Clinical Trials Partnership and the Global Fund [40] are also collaborating in different projects related to HIV, tuberculosis, and malaria, and these organizations are largely responsible for the important degree of collaboration between European and sub-Saharan African countries [41]. Research for operational health services is necessary to improve the distribution and accessibility of pneumonia treatments, including antibiotics in primary healthcare centers and oxygen in hospitals. Likewise, new vaccines still need to be developed for strains of pneumococcus that current multivalent conjugate vaccines do not protect against [8].

In addition to programs focused on financing and implementing collaborative North-South and South-South projects, other efforts could be directed toward reducing obstacles associated with publication processes that limit the dissemination of LMICs through the main international scientific journals. The literature has described obstacles related to linguistic skills and methodological deficiencies, which highlights the need to improve these areas in particular [42, 43]. Other authors have pointed to the costs associated with publishing in open access journals, so it is worth assessing whether the programs to support open access publishing implemented at an institutional level and by publishers such as PLOS, Biomed Central, or *The Lancet* Journals, are sufficient [44–46].

With regard to the impact of research, although Europe and North America are balanced in terms of the absolute number of citations, North America holds an advantage in terms of the citation rate. Research from sub-Saharan Africa also has a very high citation rate, which almost reaches that achieved in Europe. The fact that these African countries present a high degree of collaboration with researchers in the USA and Europe, who represent the “mainstream” international research interests, could help explain the high citation rates seen in this region. On the other hand, Latin America & Caribbean, South Asia, and East Asia & Pacific are all regions with generally lower citation rates, although this difference is not so pronounced in the case of papers produced in collaboration, as reported elsewhere [47].

By country, the hegemony of the USA and several European countries in terms of the number of citations received was evident, as was the lower ranking of some Asian countries, such as Japan and China, in relation to their scientific production. The positioning of China as a reference for scientific production and participation in international research networks does not correspond to its ranking with regard to citation indicators, despite their improved standing over the past several years [30]. On these indicators, China still lags behind the USA as well as the leading European countries, Canada, Australia and even nearby countries such as Japan. For now at least, the countries that have traditionally occupied the “mainstream” of scientific research still maintain their hegemony [48].

As with the relative indicators of scientific production adjusted for economic and demographic parameters, some countries surpass the major scientific systems with regard to the citation rate, which links the degree of citation with the volume of scientific production [33]. These countries may have participated in certain highly relevant contributions, or they may be small countries with highly developed scientific systems, such as Vietnam, Switzerland, South Africa, New Zealand, and Saudi Arabia. These countries also stand out for their high levels of international collaboration, which is a factor associated with more citations.

The high mean citations received by publications produced in sub-Saharan Africa, and the participation of different emerging countries like Vietnam and South Africa in some of the highest cited papers we identified, underlines the capacity of these countries to contribute to high-impact and excellent-quality scientific studies. This result is consistent with previous studies that have also demonstrated these countries’ capacity to participate in emerging research topics [49]. These specialists therefore represent an excellent asset, strengthening the human capital from high-income countries and enabling the advancement of research [50, 51].

In general, the most prestigious journals show a greater concentration of research from the USA and Europe, with greater collaboration and impact when countries from other geographical regions also participate [52].

Bacterial pneumonia is the main branch for the multidisciplinary and multipathological MeSH of “Pneumonia”, with the main areas of interest (“Community-acquired Infections”, “Anti-bacterial Agents” and “Treatment Outcome”) reflecting the focus of research on identifying risk factors and assessing different treatments and their outcomes. In publications pertaining to the MeSH “Pneumonia, Ventilator-Associated,” the main axes of the subject content according to the MeSH terms were the group of epidemiological studies and clinical and treatment protocols in intensive care. “Pneumonia Pneumocystis,” is closely related to infection due to HIV and immunodepression. The main areas of research interest for “Pneumonia, Viral,” were the epidemiological aspects related to the setting for the infection (“Community-acquired Infections” and “Hospitalization”) along with the viruses responsible (“Influenza, Human” and “Orthomyxoviridae Infections”). Finally, for the MESH “Pneumonia, Aspiration” the main research focus is “Deglutition Disorder”.

The main limitation of this present study is its analysis of only the documents included in the WoS databases and MEDLINE (80% of the documents). Thus, a number of papers were excluded from the study, particularly those written in languages other than English, as well as the proceedings included in WoS, as our searches were based on the journals included in MEDLINE. On the other hand, our approach also allowed us to precisely characterize collaboration in the area, as only recently has MEDLINE begun to include all the institutional affiliations of the authors. We were also able to analyze the citations of the publications, with a focus on the journals with the highest impact and dissemination at an international level [28].

In conclusion, pneumonia research increased steadily over the 15-year study period, with Europe and North America leading scientific production. About a fifth of all papers reflected international collaborations, and these were most evident in papers from sub-Saharan Africa and South Asia.

## Additional file

Additional file 1: **Table S1.** Descriptors included under the MeSH “Pneumonia” in PubMed. **Table S2.** Countries by regions according to World Bank Country and Lending Groups. **Table S3.** Countries by incomes according to World Bank Country and Lending Groups. **Table S4.** Top 30 countries ranked by total number of publications by quinquennium 2001–2005, 2006–2010 and 2011–2015. **Table S5.** Top 30 countries and world regions ranked according to according to population index, GDP index, GNI per capita index, R&D expenditure index and Researchers in R&D Index. **Table S6.** Top 30 countries ranked according to citations, citation rate and h-Index in the period 2001–2015. **Table S7.** Top 30 journals with the highest number of pneumonia articles published in 2001–2015, citations, citation rate (CR), impact factors for the year 2015, journal category with ranking from the Journal Citation Report and language of publication. **Table S8.** Top 30 journals with citations and citations rate (CR). **Table S9.** Top 30 citations rate (CR) journal *. **Table S10.** The 30 top general Medical Subject Headings (MeSH). **Table S11.** Top 30 countries in crude numbers of retrieved articles in “Pneumonia, Aspiration”, “Pneumonia, Bacterial”, “Pneumonia Pneumocystis”, “Pneumonia, Ventilator-Associated”, and “Pneumonia, Viral” MeSH. **Figure S1**. Evolution of scientific production on pneumonia (2001–2015). **Figure S2.** Density equalizing mapping projections. Number of documents per quinquennium for scientific production on pneumonia, (A) 2001–2005; (B) 2006–2010, and (C) 2011–2015. **Figure S3.** Density equalising mapping projections: number of documents and world development indicators, (A) GNI per capita index; (B) GDP index. **Figure S4**. Density equalising mapping projections: number of documents and world development indicators (A) population index; (B) R&D expenditure index. **Figure S5.** Top 15 journals producing the most research on pneumonia, plus citation rates. (DOCX 7194 kb)

## Data Availability

All data used to perform the study, including the information downloaded from the database as well as that derived from the treatment of the bibliographic entries, are available in the Dataverse Project, an open access public repository [23] (https://dataverse.harvard.edu/, doi: 10.7910/DVN/02BUNE).
