# Supplementary material for: A snapshot of pneumonia research activity and collaboration patterns (2001–2015): a global bibliometric analysis
Source: BMC Med Res Methodol. 2019 Sep 5;19:184. doi: 10.1186/s12874-019-0819-4 (PMC6727334; doi:10.1186/s12874-019-0819-4)
Supplement: Supplementary file 1 — Table S1. Descriptors included under the MeSH “Pneumonia” in PubMed. Table S2. Countries by regions according to World Bank Country and Lending Groups. Table S3. Countries by incomes according to World Bank Country and Lending Groups. Table S4. Top 30 countries ranked by total number of publications by quinquennium 2001–2005, 2006–2010 and 2011–2015. Table S5. Top 30 countries and world regions ranked according to according to population index, GDP index, GNI per capita index, R&D expenditure index and Researchers in R&D Index. Table S6. Top 30 countries ranked according to citations, citation rate and h-Index in the period 2001–2015. Table S7. Top 30 journals with the highest number of pneumonia articles published in 2001–2015, citations, citation rate (CR), impact factors for the year 2015, journal category with ranking from the Journal Citation Report and language of publication. Table S8. Top 30 journals with citations and citations rate (CR). Table S9. Top 30 citations rate (CR) journal *. Table S10. The 30 top general Medical Subject Headings (MeSH). Table S11. Top 30 countries in crude numbers of retrieved articles in “Pneumonia, Aspiration”, “Pneumonia, Bacterial”, “Pneumonia Pneumocystis”, “Pneumonia, Ventilator-Associated”, and “Pneumonia, Viral” MeSH. Figure S1. Evolution of scientific production on pneumonia (2001–2015). Figure S2. Density equalizing mapping projections. Number of documents per quinquennium for scientific production on pneumonia, (A) 2001–2005; (B) 2006–2010, and (C) 2011–2015. Figure S3. Density equalising mapping projections: number of documents and world development indicators, (A) GNI per capita index; (B) GDP index. Figure S4. Density equalising mapping projections: number of documents and world development indicators (A) population index; (B) R&D expenditure index. Figure S5. Top 15 journals producing the most research on pneumonia, plus citation rates. (DOCX 7194 kb) [file 12874_2019_819_MOESM1_ESM.docx]

**Supplementary Material**

**A snapshot of Pneumonia research activity and collaboration patterns (2001–2015): a global bibliometric analysis**

**Running title: Bibliometric analysis of pneumonia research**

José M. Ramos ^1 *^, Héctor Pinargote ^1^, Isabel Belinchón ^2^, Gregorio González-Alcaide^3^

^1^ Department of Internal Medicine, General University Hospital of Alicante, Alicante & Department of Clinical Medicine, Miguel Hernandez University of Elche de Elche, Alicante

^2^ Department of Dermatology, General University Hospital of Alicante, Alicante & Department of Clinical Medicine, Miguel Hernandez University of Elche de Elche, Alicante

^3^ Department of History of Science and Documentation. University of Valencia, Spain.

Table S1. Descriptors included in the MesH “Pneumonia”

All MeSH Categories

Diseases Category

Respiratory Tract Diseases

Lung Diseases

Pneumonia

Bronchopneumonia

Pleuropneumonia

Pneumonia, Aspiration (1967)

Pneumonia, Lipid

Pneumonia, Bacterial (1995)

Chlamydial Pneumonia

Pneumonia of Calves, Enzootic

Pneumonia of Swine, Mycoplasmal

Pneumonia, Mycoplasma

Pneumonia, Pneumococcal

Pneumonia, Rickettsial

Pneumonia, Staphylococcal

Pneumonia, Necrotizing (2017)

Pneumonia, Pneumocystis (2004)

Pneumonia, Ventilator-Associated (2007)

Pneumonia, Viral (1994)

In bracket years of inclusion

Table S2. Countries by regions*

| **East Asia and Pacific (38)** |  |  |
| --- | --- | --- |
| American Samoa | Korea, Rep. | Philippines |
| Australia | Lao PDR | Samoa |
| Brunei Darussalam | Macao SAR, China | Singapore |
| Cambodia | Malaysia | Solomon Islands |
| China | Marshall Islands | Taiwan, China |
| Fiji | Micronesia, Fed. Sts. | Thailand |
| French Polynesia | Mongolia | Timor-Leste |
| Guam | Myanmar | Papua New Guinea |
| Hong Kong SAR, China | Nauru | Tonga |
| Indonesia | New Caledonia | Tuvalu |
| Japan | New Zealand | Vanuatu |
| Kiribati | Northern Mariana Islands | Vietnam |
| Korea, Dem. People's Rep. | Palau |  |
| **Europe and Central Asia (n=58)** |  |  |
| Albania | Gibraltar | Norway |
| Andorra | Greece | Poland |
| Armenia | Greenland | Portugal |
| Austria | Hungary | Romania |
| Azerbaijan | Iceland | Russian Federation |
| Belarus | Ireland | San Marino |
| Belgium | Isle of Man | Serbia |
| Bosnia and Herzegovina | Italy | Slovak Republic |
| Bulgaria | Kazakhstan | Slovenia |
| Channel Islands | Kosovo | Spain |
| Croatia | Kyrgyz Republic | Sweden |
| Cyprus | Latvia | Switzerland |
| Czech Republic | Liechtenstein | Tajikistan |
| Denmark | Lithuania | Turkey |
| Estonia | Luxembourg | Turkmenistan |
| Faroe Islands | Macedonia, FYR | Ukraine |
| Finland | Moldova | United Kingdom |
| France | Monaco | Uzbekistan |
| Georgia | Montenegro |  |
| Germany | Netherlands |  |
| **Latin America & the Caribbean (n=42)** |  |  |
| Antigua and Barbuda | Curacao | Paraguay |
| Argentina | Dominica | Peru |
| Aruba | Dominican Republic | Puerto Rico |
| Bahamas, The | Ecuador | Sint Maarten (Dutch part) |
| Barbados | El Salvador | St. Kitts and Nevis |
| Belize | Grenada | St. Lucia |
| Bolivia | Guatemala | St. Martin (French part) |
| Brazil | Guyana | St. Vincent and the Grenadines |
| British Virgin Islands | Haiti | Suriname |
| Cayman Islands | Honduras | Trinidad and Tobago |
| Chile | Jamaica | Turks and Caicos Islands |
| Colombia | Mexico | Uruguay |
| Costa Rica | Nicaragua | Venezuela, RB |
| Cuba | Panama | Virgin Islands (U.S.) |
| **Middle East and North Africa (n = 21)** |  |  |
| Algeria | Jordan | Qatar |
| Bahrain | Kuwait | Saudi Arabia |
| Djibouti | Lebanon | Syrian Arab Republic |
| Egypt, Arab Rep. | Libya | Tunisia |
| Iran, Islamic Rep. | Malta | United Arab Emirates |
| Iraq | Morocco | West Bank and Gaza |
| Israel | Oman | Yemen, Rep. |
| **North America (n=3)** |  |  |
| Bermuda | Canada | United States |
| **South Asia (n=8)** |  |  |
| Afghanistan | India | Pakistan |
| Bangladesh | Maldives | Sri Lanka |
| Bhutan | Nepal |  |

(https://datahelpdesk.worldbank.org/knowledgebase/articles/906519).

Table S3. Countries by incomes*

| **Low-income countries (n=34)** |  |  |
| --- | --- | --- |
| Afghanistan | Guinea-Bissau | Sierra Leone |
| Benin | Haiti | Somalia |
| Burkina Faso | Korea, Dem. People's Rep. | South Sudan |
| Burundi | Liberia | Syrian Arab Republic |
| Central African Republic | Madagascar | Tajikistan |
| Chad | Malawi | Tanzania |
| Comoros | Mali | Togo |
| Congo, Dem. Rep | Mozambique | Uganda |
| Eritrea | Nepal | Yemen, Rep. |
| Ethiopia | Niger | Zimbabwe |
| Gambia, The | Rwanda |  |
| Guinea | Senegal |  |
| **Lower-middle income countries (n=56)** |  |  |
| Angola | Indonesia | Papua New Guinea |
| Bangladesh | Kenya | Philippines |
| Bhutan | Kiribati | São Tomé and Principe |
| Bolivia | Kosovo | Solomon Islands |
| Cabo Verde | Kyrgyz Republic | Sri Lanka |
| Cambodia | Lao PDR | Sudan |
| Cameroon | Lesotho | Swaziland |
| Congo, Rep. | Mauritania | Timor-Leste |
| Côte d'Ivoire | Micronesia, Fed. Sts. | Tunisia |
| Djibouti | Moldova | Ukraine |
| Egypt, Arab Rep. | Mongolia | Uzbekistan |
| El Salvador | Morocco | Vanuatu |
| Georgia | Myanmar | Vietnam |
| Ghana | Nicaragua | West Bank and Gaza |
| Honduras | Nigeria | Zambia |
| India | Pakistan |  |
| **Upper-middle-income countries (n=56)** |  |  |
| Albania | Fiji | Namibia |
| Algeria | Gabon | Nauru |
| American Samoa | Grenada | Paraguay |
| Armenia | Guatemala | Peru |
| Azerbaijan | Guyana | Romania |
| Belarus | Iran, Islamic Rep. | Russian Federation |
| Belize | Iraq | Samoa |
| Bosnia and Herzegovina | Jamaica | Serbia |
| Botswana | Jordan | South Africa |
| Brazil | Kazakhstan | St. Lucia |
| Bulgaria | Lebanon | St. Vincent and the Grenadines |
| China | Libya | Suriname |
| Colombia | Macedonia, FYR | Thailand |
| Costa Rica | Malaysia | Tonga |
| Cuba | Maldives | Turkey |
| Dominica | Marshall Islands | Turkmenistan |
| Dominican Republic | Mauritius | Tuvalu |
| Equatorial Guinea | Mexico | Venezuela, RB |
| Ecuador | Montenegro |  |

| **High Income countries (n=81)** |  |  |
| --- | --- | --- |
| Andorra | Germany | Oman |
| Antigua and Barbuda | Gibraltar | Palau |
| Argentina | Greece | Panama |
| Aruba | Greenland | Poland |
| Australia | Guam | Portugal |
| Austria | Hong Kong SAR, China | Puerto Rico |
| Bahamas, The | Hungary | Qatar |
| Bahrain | Iceland | San Marino |
| Barbados | Ireland | Saudi Arabia |
| Belgium | Isle of Man | Seychelles |
| Bermuda | Israel | Singapore |
| British Virgin Islands | Italy | Sint Maarten (Dutch part) |
| Brunei Darussalam | Japan | Slovak Republic |
| Canada | Korea, Rep. | Slovenia |
| Cayman Islands | Kuwait | Spain |
| Channel Islands | Latvia | St. Kitts and Nevis |
| Chile | Liechtenstein | St. Martin (French part) |
| Croatia | Lithuania | Sweden |
| Curaçao | Luxembourg | Switzerland |
| Cyprus | Macao SAR, China | Taiwan, China |
| Czech Republic | Malta | Trinidad and Tobago |
| Denmark | Monaco | Turks and Caicos Islands |
| Estonia | Netherlands | United Arab Emirates |
| Faroe Islands | New Caledonia | United Kingdom |
| Finland | New Zealand | United States |
| France | Northern Mariana Islands | Uruguay |
| French Polynesia | Norway | Virgin Islands (U.S.) |

*(https://datahelpdesk.worldbank.org/knowledgebase/articles/906519).

Table S4. Top 30 countries ranked by total number of publications by quinquennium 2001-2005, 2006-2010, and 2011-2015

| **Country** | **Total** | | *DP | **Country** | **2001-2005** | | **Country** | **2006-2010** | | **Country** | **2011-2015** | |
| --- | --- | --- | --- | --- | --- | --- | --- | --- | --- | --- | --- | --- |
|  | **N of docs** | **%** |  |  | **N of docs** | **%** |  | **N of docs** | **%** |  | **N of docs** | **%** |
| USA | 8,735 | 38.49 | -4.61 | USA | 2,248 | 41.13 | USA | 2,907 | 39.14 | USA | 3,580 | 36.52 |
| UK | 1,629 | 7.18 | 0.81 | France | 417 | 7.63 | Germany | 521 | 7.01 | China | 827 | 8.44 |
| Japan | 1,581 | 6.97 | 0.03 | UK | 403 | 7.37 | Japan | 518 | 6.97 | Japan | 725 | 7.40 |
| Germany | 1,544 | 6.80 | 0.18 | Germany | 388 | 7.10 | UK | 512 | 6.89 | UK | 714 | 7.28 |
| France | 1,527 | 6.73 | 0.30 | Japan | 338 | 6.18 | France | 498 | 6.71 | Germany | 635 | 6.48 |
| Spain | 1,251 | 5.51 | 0.81 | Spain | 297 | 5.43 | Spain | 423 | 5.70 | France | 612 | 6.24 |
| China | 1,126 | 4.96 | 0.11 | Canada | 290 | 5.31 | Canada | 361 | 4.86 | Spain | 531 | 5.42 |
| Canada | 1,091 | 4.81 | 0.74 | Netherlands | 205 | 3.75 | Italy | 298 | 4.01 | Canada | 440 | 4.49 |
| Netherlands | 911 | 4.01 | 1.43 | Italy | 160 | 2.93 | Netherlands | 279 | 3.76 | Netherlands | 427 | 4.36 |
| Italy | 859 | 3.79 | 1.35 | Australia | 150 | 2.74 | China | 237 | 3.19 | Italy | 401 | 4.09 |
| Australia | 734 | 3.23 | 1.32 | Switzerland | 128 | 2.34 | Australia | 225 | 3.03 | Australia | 359 | 3.66 |
| Brazil | 600 | 2.64 | 1.62 | Belgium | 87 | 1.59 | Brazil | 213 | 2.87 | South Korea | 315 | 3.21 |
| Switzerland | 541 | 2.38 | 1.65 | Sweden | 84 | 1.54 | Switzerland | 190 | 2.56 | Brazil | 313 | 3.19 |
| South Korea | 534 | 2.35 | 1.5 | Denmark | 83 | 1.52 | Taiwan | 149 | 2.01 | Taiwan | 296 | 3.02 |
| Taiwan | 509 | 2.24 | 0.76 | Turkey | 83 | 1.52 | South Korea | 148 | 1.99 | Switzerland | 223 | 2.28 |
| Belgium | 401 | 1.77 | 0.62 | Brazil | 74 | 1.35 | Turkey | 131 | 1.76 | Belgium | 193 | 1.97 |
| Turkey | 384 | 1.69 | 0.39 | Finland | 73 | 1.34 | Greece | 127 | 1.71 | Turkey | 170 | 1.73 |
| Sweden | 333 | 1.47 | 0.33 | South Africa | 72 | 1.32 | Belgium | 121 | 1.63 | India | 162 | 1.65 |
| India | 311 | 1.37 | 0.09 | South Korea | 71 | 1.30 | Sweden | 113 | 1.52 | Sweden | 136 | 1.39 |
| Greece | 295 | 1.30 | 0.02 | Israel | 64 | 1.17 | India | 105 | 1.41 | Greece | 117 | 1.19 |
| Denmark | 278 | 1.22 | 0 | Taiwan | 64 | 1.17 | Denmark | 80 | 1.08 | Denmark | 115 | 1.17 |
| Israel | 258 | 1.14 | 0.03 | China | 62 | 1.13 | Israel | 80 | 1.08 | Israel | 114 | 1.16 |
| South Africa | 233 | 1.03 | -0.11 | Austria | 58 | 1.06 | South Africa | 79 | 1.06 | Argentina | 93 | 0.95 |
| Finland | 217 | 0.96 | 0.01 | Greece | 51 | 0.93 | Finland | 78 | 1.05 | Austria | 92 | 0.94 |
| Austria | 205 | 0.90 | 0.03 | India | 44 | 0.81 | Argentina | 63 | 0.85 | South Africa | 82 | 0.84 |
| Argentina | 193 | 0.85 | -0.01 | Argentina | 37 | 0.68 | Austria | 55 | 0.74 | Finland | 66 | 0.67 |
| Thailand | 136 | 0.60 | 0.11 | Russia | 30 | 0.55 | Thailand | 52 | 0.70 | Poland | 65 | 0.66 |
| Chile | 125 | 0.55 | 0.17 | Mexico | 27 | 0.49 | Chile | 42 | 0.57 | Thailand | 65 | 0.66 |
| Poland | 114 | 0.50 | 0.12 | Chile | 26 | 0.48 | Ireland | 35 | 0.47 | New Zealand | 59 | 0.60 |
| Mexico | 113 | 0.50 | 0.16 | Singapore | 23 | 0.42 | New Zealand | 31 | 0.42 | Chile | 57 | 0.58 |

N of docs = numbers of documents

*DP = Difference of percentage from period 2001-2005 to period 2011-2015

Table S5. Top 30 countries and world regions ranked according to according to population index, GDP index, GNI per capita index, R&D expenditure index and Researchers in R&D Index

| **Country** | **Population**  **Index*** | **Country** | **GPD**  **Index**** | **Country** | **GNI per capita Index***** | **Country** | **R&D expenditure**  **Index ****** | **Country** | **Researchers in R&D Index******** |
| --- | --- | --- | --- | --- | --- | --- | --- | --- | --- |
| Monaco | 112.42 | Gambia. The | 30.83 | USA | 18.31 | USA | 3,276.91 | USA | 2.25 |
| Andorra | 75.86 | Malawi | 9.27 | China | 14.08 | Spain | 1,056.90 | Niger | 2.16 |
| Switzerland | 70.32 | Niger | 3.64 | India | 8.25 | UK | 993.78 | India | 1.84 |
| Netherlands | 55.23 | Uganda | 3.42 | Malawi | 5.19 | China | 735.50 | Uganda | 1.39 |
| Iceland | 51.70 | Guinea-Bissau | 2.62 | Brazil | 4.83 | Italy | 731.10 | China | 1.22 |
| Denmark | 50.54 | Andorra | 1.94 | UK | 4.67 | France | 712.03 | Malawi | 1.16 |
| Finland | 40.77 | Kenya | 1.88 | Japan | 4.54 | Germany | 589.28 | Brazil | 1.06 |
| Belgium | 37.29 | Vanuatu | 1.78 | France | 4.40 | Canada | 579.61 | Tanzania | 0.78 |
| Sweden | 35.94 | Cambodia | 1.60 | Spain | 4.20 | Brazil | 557.01 | Cambodia | 0.67 |
| Israel | 35.05 | Nepal | 1.55 | Germany | 4.06 | Turkey | 532.13 | South Africa | 0.62 |
| Australia | 34.24 | Grenada | 1.35 | Uganda | 4.04 | Netherlands | 500.78 | Italy | 0.54 |
| Canada | 32.71 | Israel | 1.26 | Bangladesh | 3.07 | Japan | 493.90 | Philippines | 0.53 |
| USA | 28.78 | Papua New Guinea | 1.26 | Canada | 2.89 | Greece | 448.47 | Colombia | 0.52 |
| Spain | 27.90 | Mozambique | 1.25 | Kenya | 2.86 | Thailand | 445.07 | Mozambique | 0.52 |
| Greece | 26.84 | Netherlands | 1.22 | Italy | 2.59 | Gambia. The | 423.33 | Turkey | 0.51 |
| UK | 26.32 | Tunisia | 1.19 | Turkey | 2.43 | India | 401.21 | Ghana | 0.50 |
| New Zealand | 25.65 | Greece | 1.18 | Niger | 2.37 | Philippines | 391.67 | Spain | 0.49 |
| Austria | 24.66 | Iceland | 1.09 | South Africa | 2.21 | Indonesia | 389.99 | Kenya | 0.45 |
| Ireland | 23.89 | Croatia | 1.07 | Netherlands | 2.16 | Colombia | 388.93 | France | 0.42 |
| France | 23.79 | Switzerland | 1.04 | Australia | 2.03 | Argentina | 386.97 | United Kingdom | 0.41 |
| Germany | 18.88 | Spain | 1.01 | Korea. Rep. | 1.93 | Chile | 347.09 | Germany | 0.41 |
| Singapore | 18.84 | Zambia | 0.96 | Gambia. The | 1.69 | Australia | 342.18 | Pakistan | 0.40 |
| Norway | 16.03 | Denmark | 0.96 | Pakistan | 1.67 | South Africa | 291.92 | Zambia | 0.37 |
| Gambia | 14.98 | Finland | 0.96 | Mozambique | 1.64 | Peru | 252.79 | Mexico | 0.36 |
| Italy | 14.62 | Belgium | 0.93 | Argentina | 1.23 | Saudi Arabia | 248.51 | Chile | 0.34 |
| Japan | 12.38 | Monaco | 0.84 | Thailand | 1.18 | Mexico | 247.87 | Ethiopia | 0.34 |
| Croatia | 12.35 | New Zealand | 0.82 | Ethiopia | 1.17 | Zambia | 221.07 | Guatemala | 0.33 |
| Luxembourg | 12.12 | South Africa | 0.82 | Greece | 1.13 | Guatemala | 197.84 | Nepal | 0.33 |
| Slovenia | 11.84 | Canada | 0.79 | Tanzania | 1.12 | Pakistan | 196.32 | Lao PDR | 0.32 |
| South Korea | 10.88 | Lao PDR | 0.79 | Nepal | 1.11 | Belgium | 195.18 | Japan | 0.30 |

* Number of publications per million population

*** *Number of publications per 1 billon US dollars of gross domestic product (GPD)*

*** Number of publications per 100 USD dollars of gross national income (GNI) per capita

**** Numbers of publications per % of GDP expenditure in Research and Development (R&D)

***** Numbers of publications per researcher per million people

Table S6. Top-30 countries ranked according by citations, citation rate (CR) and h-Index in the period 2001-2015

| **Country** | **Citations** | **Country** | **CR** | **Country** | **H index** |
| --- | --- | --- | --- | --- | --- |
| USA | 316,942 | Vietnam | 50.79 | USA | 197 |
| UK | 62,612 | Switzerland | 42.94 | UK | 106 |
| France | 48,019 | South Africa | 42.85 | France | 96 |
| Spain | 43,459 | New Zealand | 40.49 | Spain | 96 |
| Germany | 43,436 | Saudi Arabia | 38.62 | Germany | 94 |
| Canada | 40,090 | UK | 38.44 | Canada | 88 |
| Netherlands | 34,798 | Netherlands | 38.20 | Netherlands | 88 |
| Japan | 30,978 | Ireland | 36.85 | Japan | 74 |
| Italy | 25,600 | Canada | 36.75 | Switzerland | 74 |
| Switzerland | 23,228 | Sweden | 36.65 | Australia | 71 |
| Australia | 22,440 | Denmark | 36.53 | Italy | 70 |
| China | 18,370 | USA | 36.28 | Belgium | 62 |
| Belgium | 13,919 | Spain | 34.74 | Sweden | 56 |
| Sweden | 12,203 | Belgium | 34.71 | Denmark | 55 |
| Brazil | 11,136 | Finland | 34.17 | Peoples R China | 54 |
| South Korea | 10,486 | Croatia | 32.69 | Greece | 48 |
| Denmark | 10,154 | France | 31.45 | Brazil | 47 |
| South Africa | 9,985 | Kenya | 31.40 | South Korea | 47 |
| Greece | 7,851 | Czech Republic | 30.90 | South Africa | 46 |
| Taiwan | 7,424 | Australia | 30.57 | Finland | 43 |
| Finland | 7,415 | Pakistan | 30.56 | Argentina | 38 |
| Israel | 6,355 | Italy | 29.80 | Israel | 38 |
| Turkey | 5,989 | Argentina | 29.48 | Austria | 37 |
| India | 5,836 | Mexico | 29.09 | India | 36 |
| Argentina | 5,690 | Philippines | 28.25 | Taiwan | 36 |
| Austria | 5,042 | Germany | 28.13 | Turkey | 35 |
| New Zealand | 4,413 | Singapore | 27.51 | Ireland | 34 |
| Ireland | 3,832 | Bangladesh | 27.40 | New Zealand | 32 |
| Thailand | 3,685 | Thailand | 27.10 | Thailand | 30 |
| Saudi Arabia | 3,360 | Portugal | 27.04 | Portugal | 27 |

CR= citation rate

Table S7. The 30 journals with the highest number of pneumonia articles published during the period 2001-2015, citations, citation rate (CR), impact factors for the year 2015, journal category with ranking from the Journal Citation Report and language of publication

| **Top 30 journals** | **N. of docs** | **%** | **Citat.** | **CR** | **Impact factor**  **2015** | **Journal category (ranking)** | **Language** |
| --- | --- | --- | --- | --- | --- | --- | --- |
| *PLoS One* | 494 | 2.18 | 7,471 | 15.12 | 3.057 | Multidisciplinary Sciences (11 of 63) | Eng |
| *Clinical Infectious Diseases* | 412 | 1.81 | 26,351 | 63.96 | 8.736 | Immunology (9 of 151) Infectious Diseases (2 of 83) Microbiology (10 of 123) | Eng |
| *Chest* | 397 | 1.75 | 22,212 | 55.95 | 6.136 | Respiratory System (6 of 58) Critical Care Medicine (5 of 33) | Eng |
| *Journal of Immunology* | 354 | 1.56 | 17,382 | 49.10 | 4.985 | Inmunology (32 of 151) | Eng |
| *American Journal of Physiology-Lung Cellular and Molecular Physiology* | 323 | 1.42 | 11,292 | 34.96 | 4.721 | Physiology (8 of 83) Respiratory System (8 of 58) | Eng |
| *Critical Care Medicine* | 291 | 1.28 | 16,049 | 55.15 | 7.422 | Critical Care Medicine (4 of 33) | Eng |
| *European Respiratory Journal* | 283 | 1.25 | 12,025 | 42.49 | 8.332 | Respiratory System (3 of 58) | Eng |
| *Infection and Immunity* | 256 | 1.13 | 9,668 | 37.77 | 3.603 | Inmunology (56 of 151) Infectious Diseases (20 of 83) | Eng |
| *American Journal of Respiratory And Critical Care Medicine* | 256 | 1.13 | 22,647 | 88.46 | 13.118 | Critical Care Medicine (2 of 33) Respiratory System (2 of 58) | Eng |
| *American Journal of Respiratory Cell and Molecular Biology* | 251 | 1.11 | 8,225 | 32.77 | 4.082 | Biochemistry & Molecular Biology (74 of 289) Cell Biology (64 of 187) Respiratory System (10 of 58) | Eng |
| *Antimicrobial Agents and Chemotherapy* | 213 | 0.94 | 5,929 | 27.84 | 4.415 | Microbiology (22 of 123) Pharmacology & Pharmacy (34 of 255) | Eng |
| *Intensive Care Medicine* | 212 | 0.93 | 9,041 | 42.65 | 10.125 | Critical Care Medicine (3 of 33) | Eng |
| *Journal of Clinical Microbiology* | 209 | 0.92 | 6,174 | 29.54 | 3.631 | Microbiology (36 of 123) | Eng |
| *Pediatric Infectious Disease Journal* | 196 | 0.86 | 5,506 | 28.09 | 2.587 | Immunology (84 of 151) Infectious Diseases (38 of 83) Pediatrics (22 of 120) | Eng |
| *Vaccine* | 190 | 0.84 | 4,367 | 22.98 | 3.413 | Immunology (60 of 151) Medicine, Research & Experimental (36 of 124) | Eng |
| *Journal of Infectious Diseases* | 183 | 0.81 | 9299 | 50.81 | 6.344 | Immunology (20 of 151) Infectious Diseases (5 of 83) Microbiology (14 of 123) | Eng |
| *Clinical Microbiology and Infection* | 175 | 0.77 | 4,772 | 27.27 | 4.575 | Infectious Diseases (10 of 83) Microbiology (20 of 123) | Eng |
| *BMC Infectious Diseases* | 160 | 0.70 | 2,774 | 17.34 | 2.690 | Infectious Diseases (33 of 83) | Eng |
| *European Journal of Clinical Microbiology & Infectious Diseases* | 159 | 0.70 | 2,919 | 18.36 | 2.857 | Infectious Diseases (29 of 83) Microbiology (50 of 123) | Eng |
| *Thorax* | 154 | 0.68 | 9,579 | 62.20 | 8.121 | Respiratory System (4 of 58) | Eng |
| *Respiratory Research* | 153 | 0.67 | 4,410 | 28.82 | 3.751 | Respiratory System (13 of 58) | Eng |
| *Critical Care* | 148 | 0.65 | 4,780 | 32.30 | 4.950 | Critical Care Medicine (7 of 33) | Eng |
| *Pediatric Pulmonology* | 147 | 0,65 | 3,257 | 22.16 | 2.850 | Pediatrics (15 of 120) Respiratory System (23 of 58) | Eng |
| *Journal of Antimicrobial Chemotherapy* | 140 | 0.62 | 3,927 | 28.05 | 4.919 | Infectious Diseases (9 of 83) Microbiology (19 of 123) Pharmacology & Pharmacy (20 of 255) | Eng |
| *Respiratory Medicine* | 138 | 0.61 | 3,217 | 23.31 | 3.036 | Cardiac & Cardiovascular Systems (47 of 124) Respiratory System (19 of 58) | Eng |
| *Respirology* | 131 | 0.58 | 2,067 | 15.78 | 3.078 | Respiratory System (16 of 58) | Eng |
| *Internal Medicine* | 131 | 0,58 | 1,205 | 9.20 | 0.832 | Medicine, General & Internal (103 of 155) | Eng |
| *International Journal of Antimicrobial Agents* | 127 | 0.56 | 2,255 | 17.76 | 4.097 | Infectious Diseases (16 of 83) Microbiology (25 of 113) Pharmacolgy & Pharmacy (40 of 261) | Eng |
| *Scandinavian Journal of Infectious Diseases* | 126 | 0.56 | 1,819 | 14.44 | 1.366 | Infectious Diseases (66 of 83) | Eng |
| *Journal of Infection and Chemotherapy* | 123 | 0.54 | 1,107 | 9.00 | 1.425 | Infectious Diseases (62 of 83) Pharmacology & Pharmacy (198 of 255) | Eng |

N of docs = numbers of documents; Citat: = citations; CR= citation rate

Table S8. Top-30 journals with citations and citations rate (CR)

| **Journal** | **N of docs** | **Citations** | **CR** |
| --- | --- | --- | --- |
| CLINICAL INFECTIOUS DISEASES | 412 | 26,351 | 63.96 |
| AMERICAN JOURNAL OF RESPIRATORY AND CRITICAL CARE MEDICINE | 256 | 22,647 | 88.46 |
| CHEST | 397 | 22,212 | 55.95 |
| NEW ENGLAND JOURNAL OF MEDICINE | 75 | 20,860 | 278.13 |
| JOURNAL OF IMMUNOLOGY | 354 | 17,382 | 49.10 |
| CRITICAL CARE MEDICINE | 291 | 16,049 | 55.15 |
| EUROPEAN RESPIRATORY JOURNAL | 283 | 12,025 | 42.49 |
| LANCET | 54 | 11,349 | 210.17 |
| AMERICAN JOURNAL OF PHYSIOLOGY-LUNG CELLULAR AND MOLECULAR PHYSIOLOGY | 323 | 11,292 | 34.96 |
| INFECTION AND IMMUNITY | 256 | 9,668 | 37.77 |
| THORAX | 154 | 9,579 | 62.20 |
| JAMA-JOURNAL OF THE AMERICAN MEDICAL ASSOCIATION | 49 | 9,492 | 193.71 |
| JOURNAL OF INFECTIOUS DISEASES | 183 | 9,299 | 50.81 |
| INTENSIVE CARE MEDICINE | 212 | 9,041 | 42.65 |
| AMERICAN JOURNAL OF RESPIRATORY CELL AND MOLECULAR BIOLOGY | 251 | 8,225 | 32.77 |
| PLOS ONE | 494 | 7,471 | 15.12 |
| JOURNAL OF CLINICAL MICROBIOLOGY | 209 | 6,174 | 29.54 |
| EMERGING INFECTIOUS DISEASES | 98 | 6,062 | 61.86 |
| ANTIMICROBIAL AGENTS AND CHEMOTHERAPY | 213 | 5,929 | 27.84 |
| ARCHIVES OF INTERNAL MEDICINE | 61 | 5,770 | 94.59 |
| PEDIATRIC INFECTIOUS DISEASE JOURNAL | 196 | 5,506 | 28.09 |
| INFECTION CONTROL AND HOSPITAL EPIDEMIOLOGY | 109 | 4,814 | 44.17 |
| CRITICAL CARE | 148 | 4,780 | 32.30 |
| CLINICAL MICROBIOLOGY AND INFECTION | 175 | 4,772 | 27.27 |
| RESPIRATORY RESEARCH | 153 | 4,410 | 28.82 |
| VACCINE | 190 | 4,367 | 22.98 |
| ANNALS OF INTERNAL MEDICINE | 36 | 4,070 | 113.06 |
| JOURNAL OF ANTIMICROBIAL CHEMOTHERAPY | 140 | 3,927 | 28.05 |
| JOURNAL OF TRAUMA-INJURY INFECTION AND CRITICAL CARE | 98 | 3,628 | 37.02 |
| PEDIATRICS | 80 | 3,561 | 44.51 |

N of docs = numbers of documents; CR= citation rate

Table S9. Top-30 citations rate (CR) journal *

| **Journal** | **N of docs** | **Citations** | **CR** |
| --- | --- | --- | --- |
| NEW ENGLAND JOURNAL OF MEDICINE | 75 | 20,860 | 278.13 |
| LANCET | 54 | 11,349 | 210.17 |
| JAMA-JOURNAL OF THE AMERICAN MEDICAL ASSOCIATION | 49 | 9,492 | 193.71 |
| ANNALS OF INTERNAL MEDICINE | 36 | 4,070 | 113.06 |
| JOURNAL OF EXPERIMENTAL MEDICINE | 34 | 3,232 | 95.06 |
| ARCHIVES OF INTERNAL MEDICINE | 61 | 5,770 | 94.59 |
| AMERICAN JOURNAL OF RESPIRATORY AND CRITICAL CARE MEDICINE | 256 | 22,647 | 88.46 |
| PROCEEDINGS OF THE NATIONAL ACADEMY OF SCIENCES OF THE UNITED STATES OF AMERICA | 31 | 2,481 | 80.03 |
| STROKE | 35 | 2,795 | 79.86 |
| BULLETIN OF THE WORLD HEALTH ORGANIZATION | 31 | 2,278 | 73.48 |
| LANCET INFECTIOUS DISEASES | 31 | 2,135 | 68.87 |
| CLINICAL INFECTIOUS DISEASES | 412 | 26,351 | 63.96 |
| THORAX | 154 | 9,579 | 62.20 |
| EMERGING INFECTIOUS DISEASES | 98 | 6,062 | 61.86 |
| BLOOD | 48 | 2,904 | 60.50 |
| CHEST | 397 | 22,212 | 55.95 |
| CRITICAL CARE MEDICINE | 291 | 16,049 | 55.15 |
| PLOS PATHOGENS | 48 | 2,496 | 52.00 |
| JOURNAL OF INFECTIOUS DISEASES | 183 | 9,299 | 50.81 |
| JOURNAL OF VIROLOGY | 36 | 1,811 | 50.31 |
| JOURNAL OF IMMUNOLOGY | 354 | 17,382 | 49.10 |
| AMERICAN JOURNAL OF MEDICINE | 57 | 2,775 | 48.68 |
| TOXICOLOGY AND APPLIED PHARMACOLOGY | 44 | 2,127 | 48.34 |
| JOURNAL OF ALLERGY AND CLINICAL IMMUNOLOGY | 70 | 3,325 | 47.50 |
| TOXICOLOGICAL SCIENCES | 40 | 1,864 | 46.60 |
| PEDIATRICS | 80 | 3,561 | 44.51 |
| INFECTION CONTROL AND HOSPITAL EPIDEMIOLOGY | 109 | 4,814 | 44.17 |
| INTENSIVE CARE MEDICINE | 212 | 9,041 | 42.65 |
| EUROPEAN RESPIRATORY JOURNAL | 283 | 12,025 | 42.49 |
| ANESTHESIOLOGY | 46 | 1,873 | 40.72 |

* with more than 30 documents

N of docs = numbers of documents; CR= citation rate

Table S10. The 30 top general Medical Subject Headings (MeSH)

| **MesH** | **N** |
| --- | --- |
| Humans | 17,907 |
| Male | 12,600 |
| Female | 12,025 |
| Pneumonia | 10,333 |
| Middle Aged | 6,841 |
| Animals | 6,406 |
| Aged | 6,162 |
| Adult | 5,927 |
| Pneumonia, Bacterial | 4,536 |
| Anti-Bacterial Agents | 3,685 |
| Lung | 3,612 |
| Mice | 3,602 |
| Community-Acquired Infections | 3,129 |
| Aged, 80 and over | 2,987 |
| Retrospective Studies | 2,800 |
| Adolescent | 2,794 |
| Risk Factors | 2,611 |
| Child, Preschool | 2,535 |
| Child | 2,461 |
| Infant | 2,453 |
| Treatment Outcome | 2,428 |
| Prospective Studies | 2,221 |
| Bronchoalveolar Lavage Fluid | 1,955 |
| Disease Models, Animal | 1,655 |
| Cross Infection | 1,599 |
| Pneumonia, Pneumococcal | 1,593 |
| Pneumonia, Ventilator-Associated | 1,542 |
| Mice, Inbred C57BL | 1,482 |
| Time Factors | 1,468 |
| Severity of Illness Index | 1,413 |

Table S11. Top 30 countries in crude numbers of retrieved articles in “Pneumonia, Aspiration”, “Pneumonia, Bacterial”, “Pneumonia Pneumocystis”, “Pneumonia, Ventilator-Associated”, and “Pneumonia, Viral” MeSH

| **Pneumonia, Aspiration** | | **Pneumonia, Bacterial** | | **Pneumonia, Pneumocystis** | | **Pneumonia, Ventilator-Associated** | | **Pneumonia, Viral** | |
| --- | --- | --- | --- | --- | --- | --- | --- | --- | --- |
| **Country** | N | **Country** | N | **Country** | N | **Country** | N | **Country** | N |
| USA | 394 | USA | 1,709 | USA | 525 | USA | 650 | USA | 383 |
| Japan | 169 | France | 379 | France | 149 | France | 170 | China | 98 |
| Germany | 78 | Spain | 378 | UK | 106 | Spain | 139 | Japan | 95 |
| UK | 74 | Germany | 329 | Japan | 104 | Greece | 72 | UK | 83 |
| Australia | 45 | Japan | 297 | Spain | 64 | Canada | 69 | Germany | 81 |
| Canada | 44 | UK | 252 | Germany | 58 | UK | 68 | Spain | 71 |
| France | 40 | Canada | 209 | Italy | 46 | Germany | 67 | France | 66 |
| Spain | 39 | Italy | 176 | Switzerland | 38 | China | 63 | Italy | 59 |
| Turkey | 31 | Netherlands | 173 | China | 38 | Brazil | 63 | Canada | 48 |
| China | 25 | China | 171 | South Africa | 35 | Italy | 63 | Netherlands | 47 |
| Italy | 24 | Australia | 123 | Denmark | 28 | Turkey | 58 | South Korea | 41 |
| South Korea | 22 | Taiwan | 104 | Canada | 27 | Netherlands | 53 | Finland | 39 |
| Switzerland | 21 | Switzerland | 103 | Taiwan | 27 | Australia | 49 | Australia | 29 |
| Netherlands | 21 | Brazil | 100 | Netherlands | 25 | Belgium | 45 | Brazil | 26 |
| Taiwan | 21 | South Korea | 92 | Australia | 23 | India | 39 | Thailand | 21 |
| Israel | 18 | Turkey | 77 | South Korea | 23 | Argentina | 36 | Mexico | 21 |
| Belgium | 17 | Belgium | 68 | India | 21 | Taiwan | 34 | Turkey | 18 |
| India | 16 | Finland | 63 | Brazil | 21 | Japan | 25 | Saudi Arabia | 16 |
| Austria | 15 | Greece | 62 | Portugal | 19 | Switzerland | 23 | Taiwan | 16 |
| Brazil | 11 | India | 58 | Greece | 16 | Thailand | 20 | Switzerland | 16 |
| Singapore | 9 | Sweden | 53 | Israel | 16 | Austria | 16 | South Africa | 15 |
| Sweden | 8 | Israel | 52 | Sweden | 14 | Colombia | 15 | Argentina | 13 |
| Greece | 8 | Argentina | 48 | Thailand | 13 | Saudi Arabia | 15 | Belgium | 12 |
| Denmark | 7 | Denmark | 47 | Malawi | 10 | Sweden | 13 | Denmark | 12 |
| Ireland | 6 | South Africa | 45 | Turkey | 9 | Chile | 12 | Israel | 11 |
| New Zealand | 5 | Austria | 40 | Tunisia | 8 | South Korea | 12 | Chile | 11 |
| South Africa | 4 | Chile | 29 | Chile | 8 | Israel | 10 | Sweden | 10 |
| Finland | 4 | Ireland | 28 | Uganda | 7 | Tunisia | 9 | Singapore | 9 |
| Norway | 4 | Thailand | 27 | Belgium | 7 | Egypt | 9 | Austria | 9 |
| Thailand | 3 | Singapore | 22 | Russia | 7 | Portugal | 9 | Greece | 8 |

**Figure S1**. Evolution of scientific production on pneumonia (2001-2015)

**Figure S2.** Density equalizing mapping projections*. Number of documents per quinquennium for scientific production on pneumonia, (A) 2001 – 2005; (B) 2006 – 2010, and (C) 2011 – 2015

**Figure S3.** Density equalizing mapping projections*: number of documents and world development indicators, (A) GNI per capita index; (B) GDP index

.

**Figure S4**. Density equalizing mapping projections*: number of documents and world development indicators (A) population index; (B) R&D expenditure index.

*** We created cartograms using the diffusion-based method for producing density-equalizing maps (DEMP), a technique based on Gastner and Newman’s algorithm to visualize benchmarking processes by anamorphic maps [ Gastner MT, Newman MEJ. From The Cover: Diffusion-based method for producing density-equalizing maps. Proc Natl Acad Sci. 2004;101: 7499–7504.] . The cartograms were generated with ScapeToad software (version 1.1) [Andrieu D, Kaiser C, Ourednik A, The ScapeToad project. ScapeToad 1.1. Cartogram software. 2008] and QGIS (version 3.2 “[Open Source Geospatial Foundation. QGIS 3.2. Open Source Geographic Information System (GIS). 2018. ] based on a world map in ESRI Shapefile (SHP) format [24 Porto Tapiquén E. World Shapefiles [Internet]. 2015 [cited 8 Aug 2018]. Available: https://tapiquen-sig.jimdo.com/english-version/free-downloads/].

**Figure S5.** Top 15 journals producing the most research on pneumonia, plus citation rates
